# Efficacy and Mechanism of Antibiotic Resistance Gene Degradation and Cell Membrane Damage during Ultraviolet Advanced Oxidation Processes

**DOI:** 10.1021/acsestwater.4c00350

**Published:** 2024-06-05

**Authors:** Junyue Wang, Linxuan Huo, Kaiqin Bian, Huan He, Michael C. Dodd, Ameet J. Pinto, Ching-Hua Huang

**Affiliations:** † School of Civil and Environmental Engineering, Georgia Institute of Technology, Atlanta, Georgia 30332, United States; ‡ Department of Civil and Environmental Engineering, University of Washington (UW), Seattle, Washington 98195-2700, United States; § State Key Laboratory of Pollution Control and Resource Reuse, Key Laboratory of Yangtze Water Environment, Ministry of Education, College of Environmental Science and Engineering, Tongji University, Shanghai 200092, P. R. China

**Keywords:** antibiotic resistance genes, advanced oxidation process (AOP), water disinfection, chlorine, hydrogen peroxide, peracetic acid

## Abstract

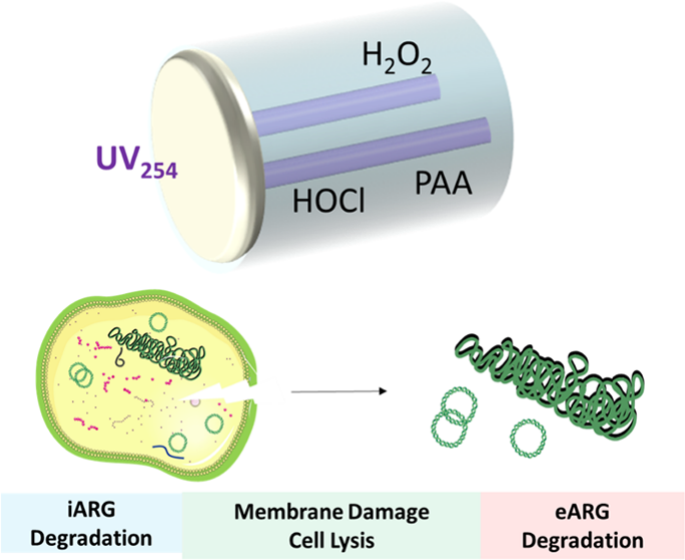

Combinations of UV with oxidants can initiate advanced oxidation processes (AOPs) and enhance bacterial inactivation. However, the effectiveness and mechanisms of UV-AOPs in damaging nucleic acids (e.g., antibiotic resistance genes (ARGs)) and cell integrity represent a knowledge gap. This study comprehensively compared ARG degradation and cell membrane damage under three different UV-AOPs. The extracellular ARG (eARG) removal efficiency followed the order of UV/chlorine > UV/H_2_O_2_ > UV/peracetic acid (PAA). Hydroxyl radical (^•^OH) and reactive chlorine species (RCS) largely contributed to eARG removal, while organic radicals made a minor contribution. For intracellular ARGs (iARGs), UV/H_2_O_2_ did not remove better than UV alone due to the scavenging of ^•^OH by cell components, whereas UV/PAA provided a modest synergism, likely due to diffusion of PAA into cells and intracellular ^•^OH generation. Comparatively, UV/chlorine achieved significant synergistic iARG removal, suggesting the critical role of the RCS in resisting cellular scavenging and inactivating ARGs. Additionally, flow cytometry analysis demonstrated that membrane damage was mainly attributed to chlorine oxidation, while the impacts of radicals, H_2_O_2_, and PAA were negligible. These results provide mechanistic insights into bacterial inactivation and fate of ARGs during UV-AOPs, and shed light on the suitability of quantitative polymerase chain reaction (qPCR) and flow cytometry in assessing disinfection performance.

## Abstract 2

This study systematically compared ARG degradation and membrane damage by three UV-advanced oxidation processes (UV/chlorine, UV/H_2_O_2_, UV/peracetic acid) and elucidated the roles of various reactive species.

## Introduction

UV-advanced oxidation processes (AOPs), i.e., simultaneous application of UV and oxidants, have been extensively explored and applied in wastewater and drinking water treatment plants (WWTPs and DWTPs).^1−3^ Notable UV-AOPs, i.e., UV/hydrogen peroxide (H_2_O_2_),^4−6^ UV/free chlorine (hereinafter referred to as UV/chlorine),^5−9^ and UV/peracetic acid (PAA),^10,11^ have been widely investigated for their high efficiency to degrade organic contaminants and inactivate pathogens, through the generation of highly reactive radicals via oxidant photolysis. However, pathogen inactivation by UV-AOPs is usually studied by culturability evaluation methods (e.g., agar plate cultivation),^12−17^ while their capabilities for genome and membrane damage are less clear and demand a better understanding. Investigating the efficiency and mechanisms of AOPs in damaging nucleic acids and cell membrane integrity is pivotal for (i) understanding the degradation and release of antibiotic resistance genes (ARGs) during AOPs, (ii) predicting the formation of viable but nonculturable (VBNC) bacteria and their potential resuscitation,^18^ and (iii) evaluating the accuracy of quantitative polymerase chain reaction (qPCR) and flow cytometry for assessing disinfection performance.

The misuse and overuse of antibiotics over the years have resulted in proliferation of ARGs and dissemination of drug-resistant diseases.^19,20^ WWTPs and DWTPs have a crucial role in ARG control within urban water cycles.^21−24^ Although disinfection processes in DWTPs and WWTPs have effectively controlled waterborne diseases by inactivation of pathogenic bacteria and viruses, ARGs are not always sufficiently degraded and could be transferred to live bacteria.^22,25−28^ Thus far, ARG degradation by common disinfection methods (i.e., ultraviolet irradiation (UV), free chlorine, monochloramine (NH_2_Cl), ozone (O_3_), and chlorine dioxide (ClO_2_)) has been extensively studied.^29−31^ It has been reported that the oxidant reactivity with both extracellular ARGs (eARGs) and intracellular ARGs (iARGs) follows the following order: O_3_ > free chlorine ≫ ClO_2_ > NH_2_Cl.^31^ Recently, PAA has been proposed as an alternative disinfectant to chlorine-based disinfectants, due to its effective bacterial inactivation and controlled disinfection byproduct formation.^32−35^ However, the reactivity of PAA toward ARGs has not been well-studied.^36,37^ So far, the performance of UV/H_2_O_2_ on ARG inactivation has been well-studied. UV/H_2_O_2_ only enhances eARG removal^31,38^ and does not lead to greater iARG removal than UV alone.^39,40^ The efficiency and mechanisms of UV/chlorine and UV/PAA for ARG control have not been fully elucidated and require more comprehensive research. UV/chlorine has been shown effective for synergistic iARG removal (relative to the sum of independent UV and chlorine disinfection);^17,41,42^ however, the eARG removal by UV/chlorine has not been studied, and the role of reactive chlorine species (RCS) in ARG removal remains controversial. UV/PAA has been reported to be effective for ARG removal on food surfaces,^43^ but its efficiency for ARG removal during wastewater treatment has been scarcely studied.

Moreover, if membrane damage and cell lysis occur during disinfection by UV-AOPs, iARGs will be released and become eARGs, which promotes ARG transfer by transformation (i.e., direct uptake of eARGs by recipient bacteria).^25,27^ Additionally, membrane damage and cell lysis cause overestimation of iARG degradation when DNA extraction is performed to recover iARG, due to overlooking eARGs resulting from iARG release. Conversely, bacterial inactivation without membrane damage could produce intact but nonculturable bacteria, posing risks of regrowth^18^ and introducing biases in flow cytometry-based pathogen monitoring (which indiscriminately counts all intact bacteria as alive).^18,44,45^

Therefore, the objective of this study was to provide fundamental chemical insights on the reactivity of radicals in three different UV-AOPs (UV/H_2_O_2_, UV/chlorine, and UV/PAA) toward nucleic acids and cell membrane and further shed light on their relative effectiveness on ARG removal and bacterial viability inactivation. In this study, we systematically compared the effects of the three UV-AOPs on the degradation of a representative ARG, ampicillin resistance gene, *bla*_TEM-1_, in extracellular and intracellular (harbored by *Escherichia coli* HB101) forms and elucidated the reactivities of different reactive species generated during each AOP. Furthermore, we examined the membrane damage effects of the UV-AOPs on a pure culture (*E. coli*) and an environmentally relevant microbial community (from a bench-scale biological activated carbon (BAC) filter) by conducting flow cytometry analyses.

## Materials and Methods

### Bacterial Cultures and Reagents

**Figure 1:**
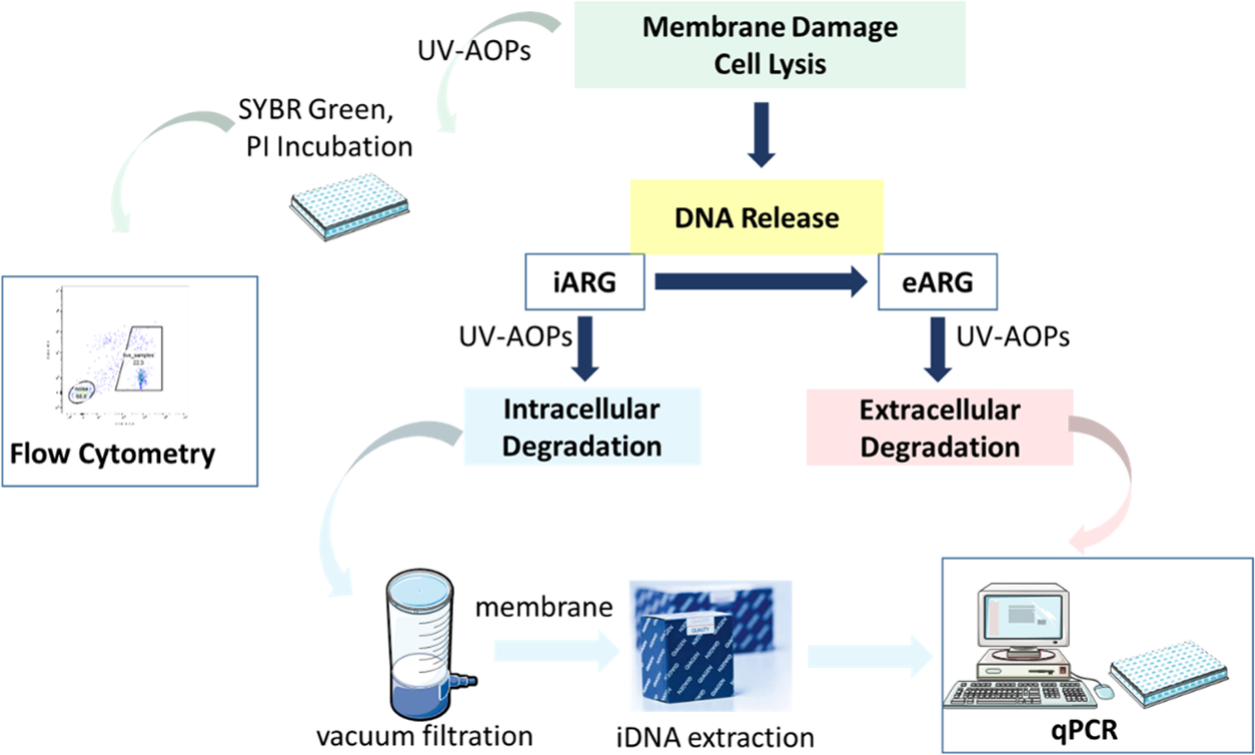
Experimental approaches for this study.

Figure 1 summarizes the overall research protocols of this study. Ampicillin resistance gene *bla*_TEM-1_ in plasmid pBR322 (Sigma-Aldrich) was used as a model eARG. *bla*_TEM-1_ in plasmid pWH1266 harbored by *Escherichia coli* (*E. coli*) HB101 (ATCC 77092) was used as a model iARG. Bacterial cells were revived according to the instructions (LB broth, 37 °C, 24 h) and washed with phosphate buffer before use. Note that the same DNA sequence was targeted for the eARG and iARG samples. Bacteria from bench-scale BAC filter effluents were collected as samples of an environmentally relevant mixed-culture community (Text S1).^46,47^ Sources of other chemicals and reagents are given in Text S1.

### Disinfection Experiments

The disinfection was conducted for UV alone, oxidant alone (H_2_O_2_, PAA, free chlorine), and UV-AOPs (simultaneous application of UV and one of the oxidants). Experiments were conducted in magnetically stirred quartz reactors containing 22 mL of the reaction solution with a light path length of 1.2 cm (i.e., the water depth). The collimated-beam UV reactor, equipped with a low-pressure UV lamp providing monochromatic light at 254 nm, was described in our previous studies (Figure S1).^13,48^ The reactor received a constant fluence rate of 9.5 ± 0.5 Einstein/(L·s) (i.e., 0.54 ± 0.03 mW/cm^2^) as measured by KI/KIO_3_ actinometry, which is relevant to the fluence rate in disinfection applications.^48,49^ Reactions were in a phosphate buffer solution (pH 7.1, 10 mM) or a synthetic source water containing natural organic matter (NOM), ions, alkalinity, and phosphate (pH 7.1, Table S1).

For eARG experiments, ∼1 × 10^10^ copies/mL of commercial pBR322 plasmid containing *bla*_TEM-1_ was added to reaction solutions. Then, experiments were undertaken by subjecting solutions to treatment with (1) an oxidant in the absence of UV light (oxidant alone), (2) an oxidant in the presence of UV light (UV-AOPs), or (3) UV light in the absence of an oxidant (UV alone). Fifty μM (i.e., 1.7 mg/L for H_2_O_2_, 3.5 mg/L for Cl_2_, and 3.8 mg/L for PAA) oxidants were used in all experiments to simulate the conditions in water disinfection applications. One-mL samples were taken at predefined intervals and quenched by 0.1 mL of Na_2_S_2_O_3_ (200 mM) to eliminate any remaining oxidants. For iARG and cell membrane damage experiments, 1 mL of bacterial culture (∼1 × 10^9^ CFU/mL *E. coli* HB101) was added in place of pBR322 plasmid, resulting in an initial cell concentration around 5 × 10^7^ CFU/mL (confirmed by plate cultivation). The whole reaction solution was then quenched with 1.0 mL of Na_2_S_2_O_3_ (200 mM) at selected time intervals for further DNA extraction and qPCR analysis.

The decay of oxidant during treating *E. coli* was evaluated by colorimetric methods using a UV–vis spectrophotometer (Text S2) based on methods reported in our previous studies.^35,50^ To quantify the relative contributions of different radicals during UV-AOP treatment, nitrobenzene (NB), diethyltoluamide (DEET), and naproxen (NPX) were used as probe compounds, and their concentration changes during AOP experiments were measured by high-performance liquid chromatography coupled to a UV diode array detector (HPLC-DAD). A mixture of 0.1% formic acid solution and acetonitrile (60:40, v/v; 0.4 mL/min) was used as the eluent. Additionally, steady-state radical concentrations were calculated by kinetic model simulations (see later discussions).

### DNA Extraction

eARG samples were added onto qPCR plates without further extraction or purification (Figure 1).^31^ For iARG samples, 5.0 mL aliquots were diluted and filtered through 0.22 μm polycarbonate membranes to collect cells and iARGs. Subsequently, the cells retained on the membranes were extracted into 0.1 mL of buffer by the DNeasy PowerSoil Pro Kits (Qiagen) for qPCR analysis (Figure 1, Text S3).

### qPCR Analysis

DNA samples were analyzed by qPCR on a StepOnePlus real-time PCR instrument (Applied Biosystems, Foster City, CA) (Text S4, Table S2), where each well contained 10 μL of sample, 3 μL of primer (resulting in a final concentration of 0.7 μM), and 7 μL of SsoAdvanced Universal SYBR Green Supermix (Bio-Rad, Hercules, CA). A standard curve is provided in Figure S2, and we found that the Na_2_S_2_O_3_ in the samples did not affect qPCR results. The employed primers were first designed by Chang et al.^51^ and targeted a 209 bp DNA segment (acquired from Integrated DNA Technologies). ARG degradation was fit to a pseudo-first-order model (eqs 1 and 2).
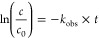
1
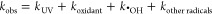
2*c* is the concentration of the ARG (in copies/mL); *c*_0_ is the initial ARG concentration; *t* is reaction time (in min); and *k*_obs_ is the observed first-order reaction rate constant (in min^–1^), which could be attributed to UV (*k*_UV_, in min^–1^), oxidant (*k*_oxidant_, in min^–1^), hydroxyl radical (^•^OH, *k*_•OH_, in min^–1^), and other radicals (*k*_other radicals_, in min^–1^). *k*_UV_ and *k*_oxidant_ could be calculated by eq 1 based on ARG degradation under UV alone and under oxidant alone. *k*_•OH_ could be calculated by multiplying the second-order rate constant between ^•^OH and selected DNA sequence (*k*_•OH,ARG_, in M^–1^min^–1^) with its steady-state concentration ([^•^OH]_ss_, in M, kinetically modeled, and probed by NB or DEET, Table 1). *k*_other radicals_ was then calculated by subtracting the other three terms (*k*_UV_, *k*_oxidant_, *k*_•OH_) from *k*_obs_.

**Table 1:** Experimental and Simulated Steady-State Hydroxyl Radical Concentration ([^•^OH]_ss_) under Different AOP Conditions (in 10^–13^ M)^a^

		UV/H_2_O_2_	UV/chlorine	UV/PAA
1	phosphate buffer (10 mM)	1.29 ± 0.12	0.74 ± 0.06	0.19 ± 0.05
		1.42*	0.50*	0.22*
2	phosphate buffer (10 mM) w/ DNA	1.15 ± 0.12	0.83 ± 0.14	0.18 ± 0.08
3	phosphate buffer (10 mM) w/ NPX	0.47 ± 0.21	0.48 ± 0.21	0.14 ± 0.02
		0.42*	0.39*	0.15*
4	synthetic source water	0.23 ± 0.01	0.28 ± 0.06	0.14 ± 0.01
		0.20*	0.24*	0.14*
6	synthetic source water w/ DNA	0.25 ± 0.05	0.36 ± 0.08	0.12 ± 0.03

a Note: Experimental conditions: [oxidants]_0_ = 50 μM (50 μM PAA contains 20 μM coexistent H_2_O_2_), UV fluence rate = 9.5 ± 0.5 Einstein/(L·s) = 0.54 ± 0.03 mW/cm^2^, pH = 7.1, temperature = 23 ± 2 °C. NB (for UV/H_2_O_2_, UV/chlorine) or DEET (for UV/PAA) was used as the probe (5 μM) for [^•^OH]_ss_ determination, [NPX]_0_ = 5 μM (added as an independent group of experiments, not together with the DNA). Synthetic source water composition is described in Table S1. *Calculated by kinetic model simulation—all the buffers and probes are included in the kinetic modeling.

### Flow Cytometry

Flow cytometry analysis was conducted to determine the decrease of intact cell counts (ICC) during UV-AOP treatment of the *E. coli* or BAC community. One-mL samples were diluted 10 times and stained with SYBR Green I (SGI, 12 μL/mL) and propidium iodide (PI, 3 μM, diluted in Tris-HCl (pH 8.5)). After staining, the samples were incubated in the dark at 37 °C for 10 min and analyzed on a Cytoflex S Flow Cytometer. The ICC degradation was fit to a pseudo-first-order model (eqs 3 and 4).
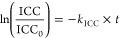
3
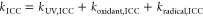
4

ICC is the concentration of the intact cells (in cells/mL); ICC_0_ is the initial intact cell concentration; *t* is reaction time (in min); *k*_ICC_ is the observed first-order reaction rate constant (in min^–1^), which could be attributed to UV (*k*_UV,ICC_, in min^–1^), oxidant (*k*_oxidant,ICC_, in min^–1^), and radicals (*k*_radical,ICC_, in min^–1^). Similarly, *k*_UV,ICC_ and *k*_oxidant,ICC_ could be calculated by eq 3 based on the ICC decrease under UV alone and oxidant alone, while *k*_radical,ICC_ was calculated by subtracting the other two terms from *k*_ICC_.

### Kinetic Simulation

The concentration of radicals during AOPs was modeled by Kintecus 4.55.31 with the reactions listed in Table S3. The photochemistry calculation was illustrated in Text S5.

## Results and Discussion

### eARG Degradation

**Figure 2:**
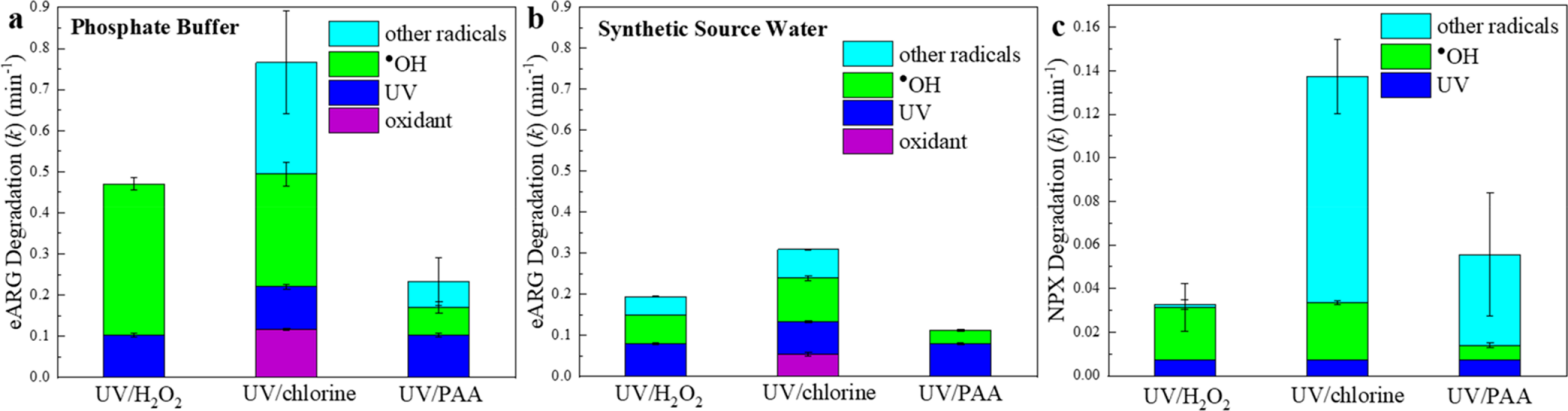
Relative contributions of oxidants (H_2_O_2_, free chlorine, PAA), UV light, and radicals to pseudo-first-order rate constants for eARG degradation in 10 mM phosphate buffer (a) and synthetic source water (b) and for NPX degradation in 10 mM phosphate buffer (c), during UV/H_2_O_2_, UV/chlorine, and UV/PAA treatments. Experimental conditions: [oxidants]_0_ = 50 μM (50 μM PAA contains 20 μM coexistent H_2_O_2_), UV fluence rate = 9.5 ± 0.5 Einstein/(L·s) = 0.54 ± 0.03 mW/cm^2^, [eARG] _0_ = ∼1 × 10^10^ copies/mL, [NPX]_0_ = 5 μM (added as an independent group of experiments, not together with the DNA), pH = 7.1, temperature = 23 ± 2 °C. Error bars represent standard deviation between duplicate experiments. The kinetic data is provided in Figure S3. The contribution from radicals is considered as synergistic inactivation by combining UV and oxidants. The contribution from ^•^OH is calculated from the measured [^•^OH]_ss_ in the presence of DNA (Table 1).

eARGs are an important contributor to environmental antibiotic resistance.^52,53^ Investigating eARG degradation in (waste)water treatment processes could provide insights into ARG removal in treatment plants as well as broaden a fundamental understanding of the reactivity of naked DNA toward reactive species. The degradation of the eARG-containing pBR322 plasmid under three UV-AOPs was studied in phosphate buffer and synthetic source water, and the contribution of different reactive species was probed and computationally simulated. Overall, the eARG degradation efficiency followed the order: UV/chlorine > UV/H_2_O_2_ > UV/PAA, regardless of the background water matrix (Figure 2); the contributions of reactive species in each process are discussed below.

For eARG degradation in phosphate buffer, a rate constant of 0.11 ± 0.01 min^–1^ was measured for degradation by UV alone (Figures S3 and 2a), which equals 0.0035 cm^2^/mJ and is comparable with Chang et al.’s results for the same amplicon.^51^ H_2_O_2_ and PAA alone resulted in negligible eARG removal, which was consistent with their low reactivity with nucleotides and RNA.^35,54^ Free chlorine at a concentration of 3.55 mg/L as Cl_2_ resulted in a measured pseudo-first-order rate constant of 0.13 ± 0.01 min^–1^ (Figures S4 and 2a), which is similar to the degradation of ∼200-bp amplicons in previous studies (∼0.8 log removal at 50 mg·min/L exposure).^39,55,56^ However, a second-order rate constant was not directly calculated because ARG degradation by free chlorine has been reported to follow complex kinetics due to a postulated sequence of reactions involving N-chlorination followed by C-chlorination (with the former step reversible by Na_2_S_2_O_3_, which was used here to quench excess free chlorine residuals upon sampling).^31,56^

The steady-state ^•^OH concentration ([^•^OH]_ss_) in the UV/H_2_O_2_ and UV/chlorine systems was calculated based on degradation of NB under these UV-AOPs, as NB is a suitable probe compound that selectively reacts with ^•^OH but not with other radicals such as RCS, or with H_2_O_2_ or free chlorine.^6,57,58^ [^•^OH]_ss_ in UV/PAA was calculated based on experimental degradation of DEET, which has been confirmed to be reactive to ^•^OH but nonreactive with organic radicals from PAA in our unpublished study. ^•^OH is the major reactive species in the UV/H_2_O_2_ system. Thus, the reactivity of ^•^OH toward the targeted ARG sequence was determined in the UV/H_2_O_2_ system, where [^•^OH]_ss_ of (1.15 ± 0.12) × 10^–13^ M (Table 1) yielded a pseudo-first-order rate constant of 0.37 ± 0.02 min^–1^, corresponding to a second-order rate constant of *k*_•OH,ARG_ = 5.36 × 10^10^ M^–1^ s^–1^ (Figure 2a). The above derived *k*_•OH,ARG_ value for the targeted eARG is comparable to reported constants for similar-sized DNA sequences (e.g., 5.9 × 10^10^ M^–1^ s^–1^ for a 266-bp sequence^31^ and 8.1 × 10^9^ M^–1^ s^–1^ for a 192-bp sequence^38^) and is employed in this study to predict the contribution of ^•^OH to eARG degradation in UV/chlorine and UV/PAA. Unlike UV/H_2_O_2_, UV/chlorine produces both ^•^OH and RCS (Table S3). This study, for the first time, demonstrates that UV/chlorine provides greater eARG removal than UV/H_2_O_2_ at equivalent concentrations of free chlorine and H_2_O_2_ despite a lower ^•^OH level during UV/chlorine treatment (Figure 2a and Table 1). The significant contribution of RCS (i.e., Cl^•^, Cl_2_^•^^–^, and ClO^•^) indicates their high reactivity with DNA molecules. UV/PAA produces both ^•^OH and organic radicals (i.e., CH_3_C(O)O^•^, CH_3_C(O)OO^•^) as reactive species (Table S3).^10,11,13^ However, organic radicals in UV/PAA appeared to contribute negligibly to ARG removal (Figure 2a). Note that kinetic modeling (Zhang et al.^11,13^ and Table S3) of UV/PAA indicate the presence of high amounts of organic radicals (e.g., [CH_3_C(O)OO^•^]_ss_ ≈ 10^–9^ M). Furthermore, the organic radicals contributed significantly to NPX removal (Figure 2c), suggesting their low reactivity toward bacterial DNA and high selectivity in degrading certain organic pollutants.^10^ Thus, the relative ineffectiveness of UV/PAA in eARG removal appears to be due to its dramatically lower ^•^OH level compared to those of UV/H_2_O_2_ and UV/chlorine (Table 1).

eARG removal in the synthetic source water matrix was slower than that in phosphate buffer for all disinfection processes (Figures S4 and 2b). The probe compound (NB) and kinetic modeling both indicated a significant decrease in ^•^OH concentration due to scavenging by the background matrix (Table 1). Overall, regardless of the background matrix, the contribution of RCS in UV/chlorine led to the most effective eARG removal by this process, while low ^•^OH concentrations and ineffectiveness of organic radicals rendered UV/PAA the least efficient for eARG removal. Noteworthy, UV alone performed relatively similarly in phosphate buffer and synthetic source water for eARG removal. Therefore, extended UV alone exposure could be a better option than UV-AOPs where the radical scavenging by the water matrix is significant.

### iARG Degradation

**Figure 3:**
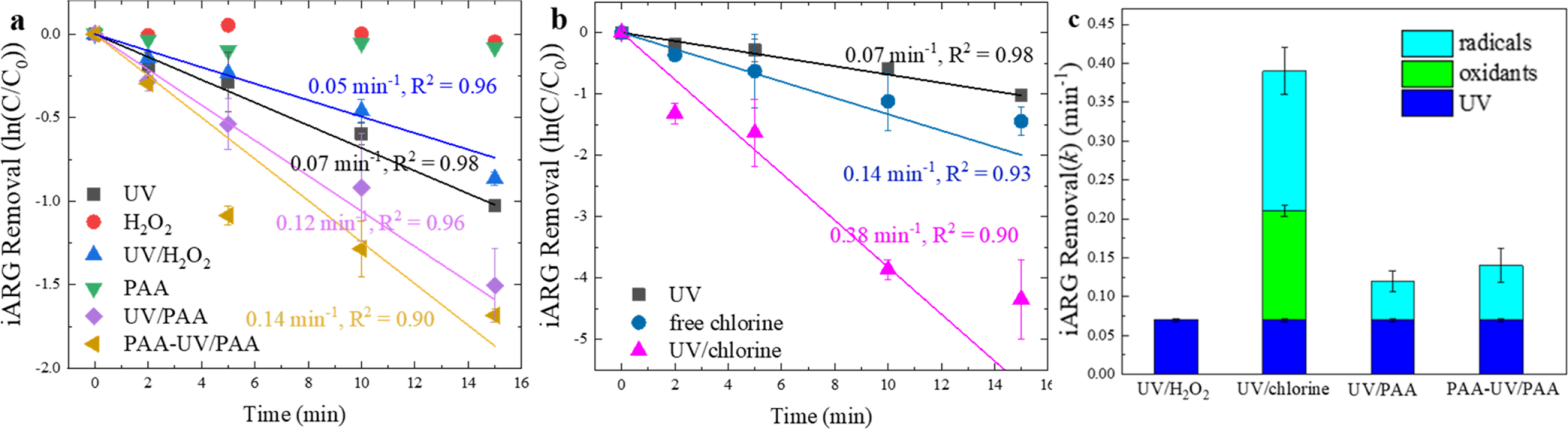
iARG degradation during disinfection by UV alone, H_2_O_2_, PAA alone, UV/H_2_O_2_, UV/PAA (a); UV alone, chlorine alone, and UV/chlorine (b); and contributions of oxidants, UV, and radicals to pseudo-first-order rate constants for iARG degradation (c). Experimental conditions: [oxidants]_0_ = 50 μM (50 μM PAA contains 20 μM coexistent H_2_O_2_), UV fluence rate = 9.5 ± 0.5 Einstein/(L·s) = 0.54 ± 0.03 mW/cm^2^, [cells]_0_ = ∼5 × 10^7^ CFU/mL, pH = 7.1, [phosphate buffer] = 10 mM, temperature = 23 ± 2 °C, pre-exposure time (to PAA) for PAA-UV/PAA = 2 min. Error bars represent standard deviation between duplicate experiments. Solid lines represent linear regression of the results (the data for H_2_O_2_ and PAA only were not analyzed due to negligible degradation). The contribution from radicals is considered as synergistic inactivation by combining UV and oxidants.

As shown in Figure 3a, UV alone led to a relatively low rate constant for iARG removal of 0.07 min^–1^, which was comparable to direct UV-degradation of eARGs in this study.^40^ H_2_O_2_ or PAA alone resulted in negligible iARG removal, consistent with their reported low reactivities toward nucleic acids.^35,54^ The combination of UV and H_2_O_2_ (*k* = 0.05 min^–1^) did not produce additional iARG removal beyond that observed for UV alone, consistent with scavenging of ^•^OH by cellular components before it could reach bacterial genomes; hence, UV/H_2_O_2_ could not achieve synergistic ARG degradation, despite the high direct reactivity of DNA molecules toward ^•^OH.^15,39,40^ In contrast, UV/PAA provided a modest synergistic effect on iARG removal (*k* = 0.12 min^–1^), where the synergism was slightly enhanced by a 2 min pre-exposure (i.e., exposure to PAA for 2 min before UV irradiation) (*k* = 0.14 min^–1^). As the organic radicals are not reactive toward DNA, the synergism of UV/PAA is tentatively attributed to penetration of PAA through the cell membrane and into the cytoplasm, where it produced ^•^OH that can more readily access the genome than those produced under UV/H_2_O_2_.^12,13,35^ As demonstrated in our previous study, H_2_O_2_ may also diffuse into the cells but is not accumulated intracellularly, probably due to enzymatic scavenging.^35,59,60^ However, the synergism in UV/PAA was limited compared to that of UV/chlorine (Figure 3b,c).

**Figure 4:**
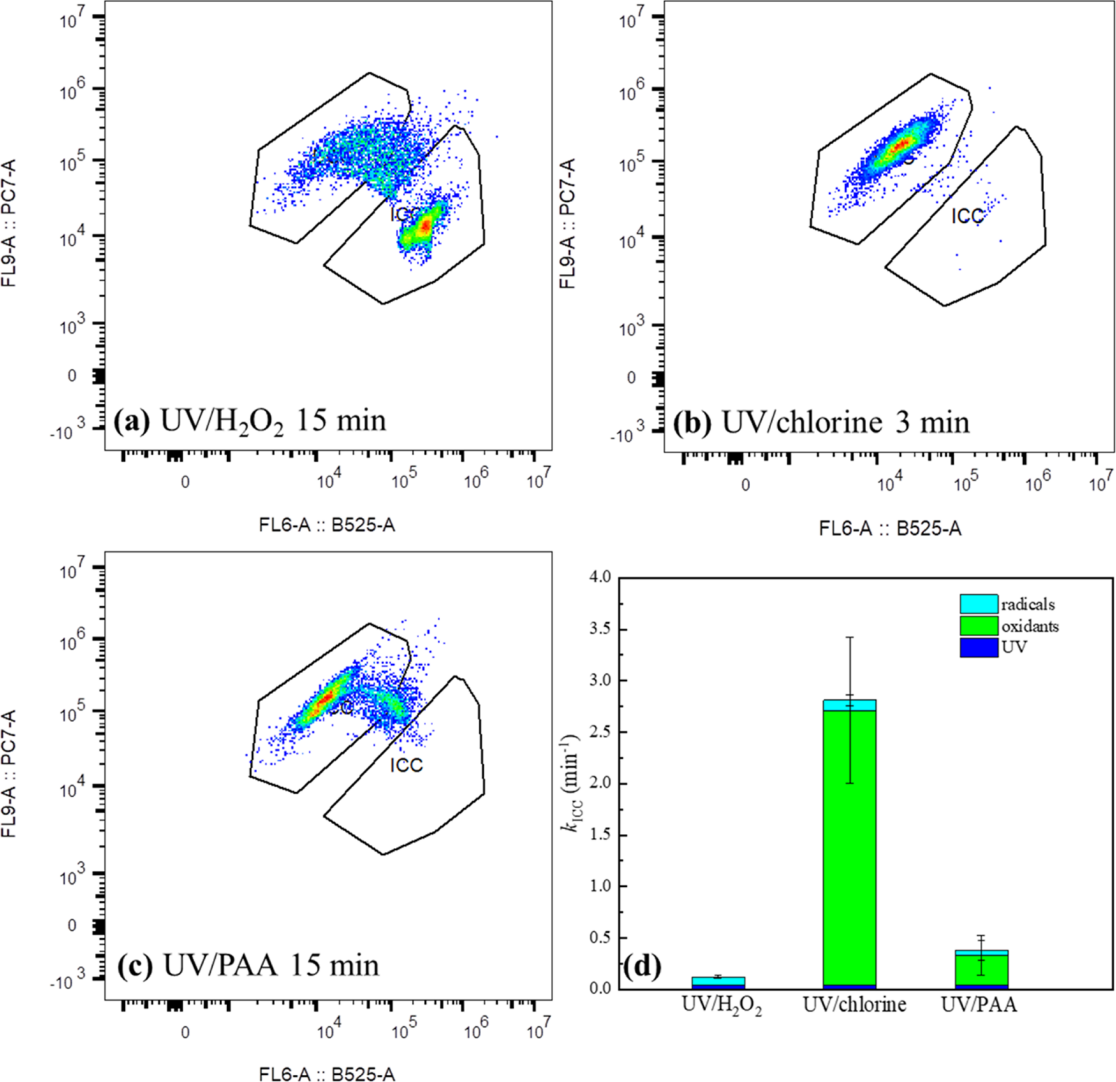
Flow cytometry dot plots of *E. coli* HB101 after UV/H_2_O_2_ (a), UV/chlorine (b), and UV/PAA (c) treatment; relative contributions of oxidants, UV light, and radicals to pseudo-first-order rate constants for degradation of intact cells in UV-AOPs (d). All samples were stained with SYBR Green and PI. The *x*-axes in panels a–c represent green fluorescence signal at 520 nm and the *y*-axes represent a red fluorescence signal at 615 nm. Electronic gates were used to separate live cells, dead cells, and background noise. Experimental conditions: [oxidants]_0_ = 50 μM (50 μM PAA contains 20 μM coexistent H_2_O_2_), [cells]_0_ = ∼5 × 10^7^ CFU/mL, UV fluence rate = 9.5 ± 0.5 Einstein/(L·s) = 0.54 ± 0.03 mW/cm^2^, pH = 7.1, [phosphate buffer] = 10 mM, temperature = 23 ± 2 °C. The contribution from radicals is considered as synergistic inactivation by combining UV and oxidants.

**Figure 5:**
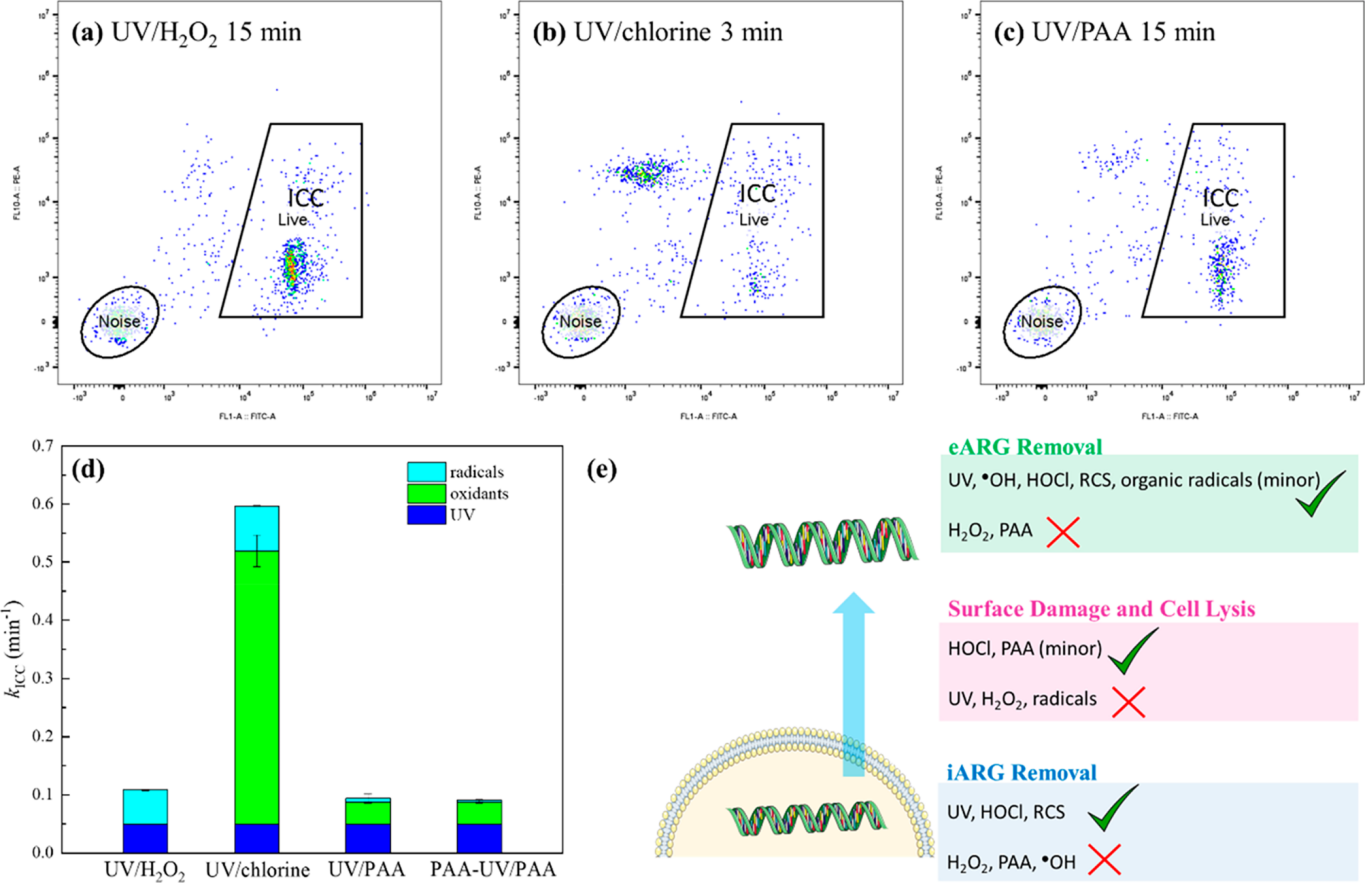
Flow cytometry dot plots of BAC bacteria community after UV/H_2_O_2_ (a), UV/chlorine (b), and UV/PAA (c) treatment; relative contributions of oxidants, UV light, and radicals to pseudo-first-order rate constants for degradation of intact cells in UV-AOPs (d); roles of different oxidants and radicals during disinfection (e). All samples were stained with SYBR Green and PI. The *x*-axes in panels a–c represent a green fluorescence signal at 520 nm and the *y*-axes represent a red fluorescence signal at 615 nm. Electronic gates were used to separate live cells, dead cells, and background noise. Experimental conditions: [oxidants]_0_ = 50 μM (50 μM PAA contains 20 μM coexistent H_2_O_2_), UV fluence rate = 9.5 ± 0.5 Einstein/(L·s) = 0.54 ± 0.03 mW/cm^2^, pH = 7.1, [phosphate buffer] = 10 mM, temperature = 23 ± 2 °C, pre-exposure time (to PAA) for PAA-UV/PAA = 2 min. The contribution from radicals is considered as synergistic inactivation by combining UV and oxidants.

Free chlorine alone at a concentration of 3.55 mg/L as Cl_2_ resulted in a pseudo-first-order rate constant of 0.14 min^–1^ for iARG removal, similar to that for eARG removal (*k* = 0.13 min^–1^) (Figure 2a). However, as chlorine could lead to cell membrane damage (see later discussion) and DNA release, the iARG removal may be attributable to both iARG recovery loss and intracellular gene degradation.^16,56^ Unlike UV/H_2_O_2_ and UV/PAA, clear synergistic iARG removal was achieved by UV/chlorine (*k* = 0.38 min^–1^, an increase of 0.17 min^–1^ compared with UV alone + chlorine alone). Theoretically, free chlorine alone and UV/chlorine should result in comparable iARG release because their cell membrane damage effects were similar (Figures 4–5), where membrane damage in UV/chlorine was mainly attributed to chlorine alone, while UV and radicals only provided minor contributions (see later discussion). Therefore, the synergistic iARG removal by UV/chlorine is likely derived from intracellular ARG destruction by photogenerated radicals rather than enhanced membrane damage. As is apparent from UV/H_2_O_2_ treatment, ^•^OH is readily scavenged by cellular components and does not contribute to iARG removal (Figure 3a). In addition, our previous study included a fluorescence microscopy study and demonstrated that intracellular accumulation of free chlorine was not more significant than that of PAA due to chlorine consumption by cellular components, suggesting that the intracellular ^•^OH production by UV/chlorine should be no more significant than that of UV/PAA.^35^ Hence, it is likely that RCS, which are unique to the UV/chlorine system, are responsible for the remarkable synergistic iARG removal by UV/chlorine, through better resistance to scavenging due to higher selectivity in their reactions with cellular components. Another possibility is free chlorine induced iARG release and hence increased the accessibility of radicals (both RCS and ^•^OH) to those DNA molecules. Therefore, further investigation should be conducted to address synergistic iARG removal by UV/chlorine.

### Membrane Damage of *E. coli* HB101

The membrane damage of the *E. coli* HB101 culture during different disinfection processes was studied by flow cytometry (Figures 4, S5–S12). As shown in Figure 4a, UV/H_2_O_2_ exhibited limited efficacy for damaging the cell membrane, rendering the majority of bacteria remained in the intact region after 15 min of AOP treatment. In contrast, UV/chlorine achieved near complete elimination of intact*E. coli* cells within only 3 min (Figure 4b). The cell membrane-damaging performance of UV/PAA was lower than that of UV/chlorine, where the culture obviously moved toward the non-intact zone, while the cell damage was not complete after 15 min (indicated by the dots between dead and intact cell zones in Figure 4c).

First-order degradation rate constants, *k*_ICC_, for ICC (cells with intact membrane) were calculated for each treatment, where synergistic ICC removal during AOPs (i.e., beyond the sum of UV alone and oxidant alone) was attributed to radicals (eq 4). As shown in Figure 4d, cell membrane damage was mainly attributed to the oxidants; e.g., free chlorine and PAA contributed to *k*_oxidant,ICC_ at 2.67 ± 0.71 and 0.29 ± 0.19 min^–1^, respectively. On the other hand, UV and radicals did not lead to significant cell membrane damage (*k* < 0.1 min^–1^ for all AOPs), suggesting that UV-AOPs could not enhance membrane damage relative to single oxidant processes.

### Membrane Damage of the BAC Microbial Community

To further test the AOPs’ membrane damage capacity on environmentally relevant microbial communities, disinfection treatment of the bacteria collected from a bench-scale BAC filter was studied by flow cytometry (Figures 5, S13–S15). As shown in Figure 5a–c, large portions of bacterial cells were damaged after 3 min UV/chlorine treatment, while UV/H_2_O_2_ and UV/PAA led to limited cell surface damage after 15 min treatment, indicating the lower cell membrane-damaging capacity of the latter two AOPs. Compared with the membrane damage of the pure culture *E. coli*, the membrane damage of the environmentally relevant BAC community was significantly slower, especially for chlorine and PAA oxidation, indicating the higher vulnerability of lab-cultured *E. coli* compared to the environmental community.^47^

As shown in Figures 5d and S13, UV, H_2_O_2_, and PAA alone did not induce significant cell membrane damage of the BAC community (*k* < 0.1 min^–1^). Free chlorine alone induced significant ICC loss (*k*_oxidant,ICC_ = 0.47 ± 0.03 min^–1^), indicating the membrane damage effect of HOCl, consistent with the *E. coli* experiments and previous studies.^61−63^ Furthermore, similar to the results for *E. coli*, radicals generated by UV/H_2_O_2_, UV/chlorine, and UV/PAA only contributed to 0.06 ± 0.01, 0.08 ± 0.01, and 0.01 ± 0.01 min^–1^ of the respective overall rate constants for ICC removal by each treatment (*k*_radical,ICC_), minor contributions compared to the significant cell membrane-damaging effect of free chlorine alone (Figure 5d). Additionally, pre-exposure to PAA (PAA-UV/PAA) could not increase cell membrane damage in UV/PAA treatment of the BAC community.

As mentioned in the introduction session, cell membrane damage plays critical roles in ARG release, bacteria regrowth, and monitoring. Overall, free chlorine resulted in considerably greater levels of membrane damage than the other oxidants and radicals investigated here, indicating a greater potential for the release of iARGs and other intracellular components during treatments in which free chlorine is present (e.g., UV/chlorine). As previous studies have shown cell membrane damage could lead to cell lysis and DNA release,^16^ these results also suggest that iARG removal by chlorination may be overestimated in previous studies because the released iARGs that were not recoverable during DNA extraction could be incorrectly counted as degraded. To gain insight into ARG release by chlorination, we tried to analyze released ARG from the membrane filtrate, yet could not track the ARG concentration with high-quality data. Hence, ARG release during chlorine-based oxidation processes is worthy of future investigation.

On the other hand, neither independent or simultaneous applications of UV, H_2_O_2_, or PAA could lead to significant cell membrane damage of the BAC community. Given that PAA and UV/PAA processes could efficiently inactivate the culturability of bacteria,^12,13,35,48^ these results suggest flow cytometry-based monitoring often cannot provide an accurate means of measuring bcaterial inactivation by PAA-based disinfection processes. Nonetheless, the resuscitation potential of the intact but nonculturable bacteria after PAA-based treatment should be further investigated.

## Conclusions

This work, for the first time, systematically compared eARG degradation, iARG degradation, and membrane damage by three UV-AOPs (UV/H_2_O_2_, UV/chlorine, and UV/PAA). Among the treatments by H_2_O_2_, free chlorine, PAA, or UV light alone, free chlorine was the most effective to damage ARG and the cell membrane due to its relatively nonselective oxidation of nucleic acids, proteins, and lipids (Figure 5e). Radicals were effective for eARG removal but contributed little to iARG removal (except for UV/chlorine) and membrane damage, likely due to competitive scavenging by cellular components. In particular, the mechanisms of synergistic iARG removal by UV/chlorine merit further study; for instance, by (1) assessing the impacts of variations in DNA sequence and nucleobase content on their reactivity with RCS, (2) monitoring oxidation products via gel electrophoresis and/or mass spectrometry, and (3) investigating DNA release and its accessibility by radicals (both RCS and ^•^OH) during UV/chlorine treatment.

The impairment of cell membrane integrity is an essential prerequisite for not only inducing ARG release but also observing successful inactivation on viability-based microbial quantification instruments (i.e., dye-staining-based methods, including flow cytometry and fluorescence microscopy). The inactivated cells, even though nonculturable, may be considered alive when the analytical method switches from plate cultivation to flow cytometry. Hence, flow cytometry could not accurately interpret the disinfection by nonchlorine-based processes (e.g., UV, UV/H_2_O_2_, PAA, UV/PAA).

Recently, PAA has been applied as a replacement for chlorine to reduce disinfection byproduct formation. Despite its effective culturability inactivation,^12,35,64^ PAA exhibited limited ICC elimination and genome damage, as demonstrated by flow cytometry and qPCR, respectively. Thus, complementary UV disinfection may be warranted for PAA to ensure ARG removal and to reduce microbial regrowth risks.

## Supporting Information Available

Three supplementary texts (chemicals and analytical methods), four supplementary tables (qPCR methods, kinetic models), and supplementary figures (ARG and ICC decay, flow cytometry plots) (PDF)
