# Supplementary material for: Efficacy and Mechanism of Antibiotic Resistance Gene Degradation and Cell Membrane Damage during Ultraviolet Advanced Oxidation Processes
Source: ACS ES T Water. 2024 Jun 5;4(6):2746–55. doi: 10.1021/acsestwater.4c00350 (PMC11186015; doi:10.1021/acsestwater.4c00350)
Supplement: Supplementary file 1 — ew4c00350_si_001.pdf [file ew4c00350_si_001.pdf]

Supporting Information

Efficacy and Mechanism of Antibiotic Resistance Gene Degradation and Cell Membrane Damge during Ultraviolet Advanced Oxidation Processes

Junyue Wang,[†] Linxuan Huo,[†] Kaiqin Bian,[†] Huan He,[#]
Michael C. Dodd,[‡] Ameet J. Pinto,[†] Ching-Hua Huang*,[†]

[†]School of Civil and Environmental Engineering, Georgia Institute of Technology, Atlanta, Georgia 30332, United States

[‡]Department of Civil and Environmental Engineering, University of Washington (UW), Seattle, Washington 98195-2700, United States

[#]State Key Laboratory of Pollution Control and Resource Reuse, Key Laboratory of Yangtze Water Environment, Ministry of Education, College of Environmental Science and Engineering, Tongji University, Shanghai 200092, P.R. China

*Corresponding Author. E-mails: ching-hua.huang@ce.gatech.edu (Ching-Hua Huang)

Numbers of Pages: 37

Numbers of Texts: 5

Numbers of Tables: 3

Numbers of Figures: 16

Numbers of References: 21

Text S1. Chemicals and Reagents

Hydrogen peroxide (H_2O_2), peracetic acid (PAA), sodium hypochlorite (NaClO), sodium hydroxide (NaOH), sulfuric acid (H_2SO_4), sodium thiosulfate ($Na_2S_2O_3$), nitrobenzene (NB), diethyltoluamide (DEET), naproxen (NPX), horseradish peroxidase (HRP), 2,2'-azino-bis(3-ethylbenzothiazoline-6-sulfonic acid) (ABTS), *N*,*N*-diethyl-*p*-phenylenediamine (DPD), possasium iodide (KI), and Na_2HPO_4 were purchased from Sigma-Aldrich or Fisher Scientific (Fair Lawn, NJ). Deionized water (DI water) (> 18 mΩ-cm) was produced from a Milli-Q water purification system (Billerica, MA).

SYBR Green I (SG) was purchased from Invitrogen™ (Cat. No.: S7585), propidium iodide (PI) was acquired from Molecular Probes™ (Cat. No.: P3566), and Tris-HCl (pH 8.5) was purchased from Bioworld (Cat. No: NC1213695).

Bacteria community from effluents of a bench-scale biologically activited carbon (BAC) filter was collected by centrifuging 50 mL samples at 15000 rpm and resuspend the pellets in 5 mL clean phosphate buffer.[1,2]

Text S2. Oxidant Concentration Analysis

The oxidants loss during treatment of iARG was tested by mixing 50 µM of oxidants with ~5×10^7 CFU/mL of bacteria cells. 1-mL alioquits were collected at defined time intervals and diluted by 10 times before measurement. H_2O_2 concentration was measured by a HRP-ABTS method at 415 nm using the UV-vis spectrophotometer. Free chlorine concentration was measured by DPD method at 515 nm, while PAA was measured by KI-DPD method at 515 nm, and the detailed procedure was provided in our previous studies.[3]

As shown in Figure S4, the degradation of H_2O_2 and PAA during 15-min disinfection was negligible. However, ~35% loss of free chlorine was observed.

Text S3. DNA Extraction Methods

In this study, we adopted vacuum filtration of oxidant-, UV-, and AOP-treated cell suspensions through polycarbonate membranes (reported to have the lowest affinity to eDNA[4]), followed by the extraction by DNeasy® PowerSoil® Pro Kits of cells retained on the membrane, for iARG recovery and purification prior to analyses. Five-mL samples were diluted and the cells (containing iARGs) were collected by vacuum filtration onto 0.22 µm membranes. The cells on the membranes were subsequently extracted into 0.1-mL concentrate by the DNeasy® PowerSoil® Pro Kits (Qiagen) according to instructions.

The iARG recovery of this method could be calculated by control extraction of *E. coli* HB101-harbored pWH1266 plasmid (carrying $bla_{\text{TEM-1}}$) before disinfection. According to plating on ampicillin-containing agar, we found the cell concentration of the sample was ~5×10^7 CFU/mL. Note that we added 5-mL sample for membrane filtration and collected only 0.1 mL of extract for iARG quantification, according to instructions of PowerSoil®. Thus, the cells in our sample were concentrated 50 times (i.e., final *E. coli* concentration in the extract should be ~2.5×10^9 CFU/mL). Analyses by qPCR showed the ARG concentration in the 0.1-mL control extract was ~2×10^9 copies/mL, indicating good recovery. Nonetheless, each *E. coli* HB101 may contain multiple pWH1266 plasmids, hence the absolute recovery (i.e, recovered ARG copies/ARG copies in the samples) cannot be calculated with certainty. As an additional check, the recovery of *E. coli* genome was estimated by comparing the extracted total DNA concentration versus theoretical concentration of *E. coli* genome in the raw sample to be ~33%.[5] However, this result may not represent the plasmid recovery efficiency due to the different DNA size and conformation.

Text S4. qPCR Analysis

DNA samples were analyzed by qPCR on a StepOnePlus real-time PCR instrument (Applied Biosystems, Foster City, CA) with the termparuture profile summarized as Table S2. We used a signal threshold at 0.007 for determination of threshold cycle number (Cq) and resulted in a standard curve with a slope of -0.29 (representing qPCR efficiency) and a R^2 of 0.998 (Figure S2). Autocalved phosphate buffer (prepared with DI water) was used as the no-template control (NTC) and we found the signal was below the threshold after 40 amplification cycles.

As the NTC did not lead to amplification, the limit of detection (LOD) was arbitrarily determined as 3 copies per reaction – the lowest possible LOD for a qPCR reaction.[6,7] Therefore, considering 10 μL of samples was added per reaction, the LOD was calculated to be 300 copies/mL for this study. The lowest concentration used in the standard curve is 2.23×10^5 copies/mL and still resulted in reproducible measurement, hence limit of quantification (LOQ) for the assay should be lower than 2.23×10^5 copies/mL. All the samples collected from degradation experiments had a concentration higher than 2.23×10^5 copies/mL (Figures 3, S2).

Text S5. Photochemcial Calculations

The first-order rate constants for photolysis of oxidants (Table S3) was calculated by eq S1.

$$\frac{\mathrm{dc}}{\mathrm{dt}} = -\frac{\Phi\varepsilon\mathrm{c}}{(\alpha+\varepsilon\mathrm{c})}\left(1 - 10^{-(\alpha+\varepsilon\mathrm{c})\mathrm{d}}\right) \times \mathrm{I} \qquad \text{(S1)}$$

c is the concentration of the photophore (in M); Φ is the quantum yield (in mol/Einstein, or unitiless if we consider ensitein as moles of photons); ε is the molar absorption coefficient at the specific wavelength (in $M^{-1}cm^{-1}$); α is the absorption coefficient (in cm^{-1}) of water matrix; d is the path length, which equals the water depth in our set-up (Figrue S1) (in cm); I is the fluence rate received by the reactor at the current set-up (in Einstein/(L·s)). When the experiments were conducted in clean phosphate buffer, $\alpha = 0$ and the total absorbance was less than 0.02, hence eq S1 could be simplified to eq S2.

$$\frac{\mathrm{dc}}{\mathrm{dt}} = -2.303 \times \varepsilon \times \Phi \times \mathrm{I} \times \mathrm{d} \times c \qquad \text{(S2)}$$

When the experiments were conducted in matrix simulating source water, α was determined to be 0.12 cm^{-1} by a UV-visible spectrophotometer, which is much higher than the absorbance by oxidants (εc), and the total absorbance was less than 0.02, hence eq S1 could be simplified to eq S3.

$$\frac{\mathrm{dc}}{\mathrm{dt}} = -\frac{\Phi\varepsilon\mathrm{c}}{\alpha}\left(1 - 10^{-\alpha\mathrm{d}}\right) \times \mathrm{I} = -2.01 \times \varepsilon \times \Phi \times \mathrm{I} \times \mathrm{d} \times c \qquad \text{(S3)}$$

In addition, unit conversion can be conducted for fluence rate I by eq S4.

$$9.5 \text{ einstein/(L·s)} \times \mathrm{d} \times \mathrm{h} \times \mathrm{c} / \lambda = 0.54 \text{ mW/cm}^2 \qquad \text{(S4)}$$

where h is the Planck constant (6.626×10^{-34} J·s, c is the speed of light in vacuum (3×10^{8} m/s), λ is the photon wavelength (254 nm in this study).

Table S1: Composition of the synthetic source water

water quality parameters	concentration
Cl^-	0.1 mM
Br^-	2 μM
Alkalinity	50 mg/L as $CaCO_3$
NH_4^+-N	0.28 mg/L as N (0.02 mM)
NO_3^--N	2.8 mg/L as N (0.2 mM)
NOM	6.0 mg/L (~2.7 mg/L as C)
phosphate buffer	1 mM
pH	7.1

Table S2: Temperature profile of qPCR analyses

stage	cycle	temp	time	ref
polymerase activation, DNA denaturation	1	98 °C	2 min	Biorad mastermix instruction
denaturation	40	98 °C	5 s	Biorad mastermix instruction
annealing	40	53.8 °C	15 s	Reference of the Primers
extension	40	60 °C	15 s	Biorad mastermix instruction
melting curve	1	60 °C to 95 °C		defaulted by the instrument

Table S3: Principal reactions in the kinetic model.

No.	Reaction	Rate constant	Ref
	**Photolysis (6 reactions)**		
1	$HOCl \rightarrow HO^{\bullet} + Cl^{\bullet}$	ε_{254} = 62 $M^{-1}cm^{-1}$, Φ = 0.62	
2	$OCl^{-} \rightarrow O^{\bullet-} + Cl^{\bullet}$	ε_{254} = 60 $M^{-1}cm^{-1}$, Φ = 0.55	
3	$H_2O_2 \rightarrow 2\ HO^{\bullet}$	ε_{254} = 18.7 $M^{-1}cm^{-1}$, Φ = 0.5	
4	$NH_2Cl \rightarrow {}^{\bullet}NH_2 + {}^{\bullet}Cl$	ε_{254} = 371 $M^{-1}cm^{-1}$, Φ = 0.2	
5	$CH_3C(O)OOH \rightarrow CH_3COO^{\bullet} + HO^{\bullet}$	ε_{254} = 8 $M^{-1}cm^{-1}$, Φ = 0.88	
6	$HOBr \rightarrow HO^{\bullet} + Br^{\bullet}$	ε_{254} = 77 $M^{-1}cm^{-1}$, Φ = 0.43	
	**Reactive Oxygen Species (ROS) (18 reactions)**		
1	$H^{+} + HO_2^{-} \rightarrow H_2O_2$	5.00×10^{10} M^{-1} s^{-1}	8
2	$H_2O_2 \rightarrow H^{+} + HO_2^{-}$	1.30×10^{-1} s^{-1}	8
3	$H^{+} + OH^{-} \rightarrow H_2O$	1.00×10^{11} M^{-1} s^{-1}	8
4	$H_2O \rightarrow H^{+} + OH^{-}$	1.00×10^{-3} s^{-1}	8
5	$H^{+} + O_2^{\bullet-} \rightarrow HO_2^{\bullet}$	5.00×10^{10} M^{-1} s^{-1}	8
6	$HO_2^{\bullet} \rightarrow H^{+} + O_2^{\bullet-}$	7.00×10^{5} s^{-1}	8
7	$HO^{\bullet} + HO^{\bullet} \rightarrow H_2O_2$	5.50×10^{9} M^{-1} s^{-1}	8
8	$HO^{\bullet}$ + OH- $\rightarrow O^{\bullet-} + H_2O$	1.30×10^{10} M^{-1} s^{-1}	8
9	$HO^{\bullet} + H_2O_2 \rightarrow HO_2^{\bullet} + H_2O$	2.70×10^{7} M^{-1} s^{-1}	8
10	$HO^{\bullet} + HO_2^{-} \rightarrow HO_2^{\bullet} + OH^{-}$	7.50×10^{9} M^{-1} s^{-1}	8
11	$HO^{\bullet} + HO_2^{\bullet} \rightarrow O_2 + H_2O$	7.10×10^{9} M^{-1} s^{-1}	8
12	$HO^{\bullet} + O_2^{\bullet-} \rightarrow O_2 + OH^{-}$	1.00×10^{10} M^{-1} s^{-1}	8
13	$HO_2^{\bullet} + HO_2^{\bullet} \rightarrow H_2O_2 + O_2$	8.30×10^{9} M^{-1} s^{-1}	8
14	$HO_2^{\bullet} + O_2^{\bullet-} \rightarrow HO_2^{-} + O_2$	9.70×10^{7} M^{-1} s^{-1}	8
15	$HO_2^{\bullet} + H_2O_2 \rightarrow O_2 + HO^{\bullet} + H_2O$	3.00 M^{-1} s^{-1}	8
16	$O_2^{\bullet-} + H_2O_2 \rightarrow O_2 + HO^{\bullet} + OH^{-}$	1.30×10^{-1} M^{-1} s^{-1}	8
11	$O^{\bullet-} + H_2O \rightarrow HO^{\bullet} + OH^{-}$	1.80×10^{6} M^{-1} s^{-1}	8
18	$O^{\bullet-} + O^{\bullet-} \rightarrow O_2^{2-}$	4.65×10^{9} M^{-1} s^{-1}	9
	**ROS w/ Bicarbonate (12 reactions)**		
1	$HCO_3^{-} + H^{+} \rightarrow H_2CO_3$	5.00×10^{10} M^{-1} s^{-1}	8
2	$H_2CO_3 \rightarrow HCO_3^{-} + H^{+}$	5.00×10^{5} s^{-1}	8
3	$CO_3^{2-} + H^{+} \rightarrow HCO_3^{-}$	5.00×10^{10} M^{-1} s^{-1}	8
4	$HCO_3^{-} \rightarrow CO_3^{2-} + H^{+}$	2.50 s^{-1}	8
5	$HO^{\bullet} + CO_3^{2-} \rightarrow CO_3^{\bullet-} + OH^{-}$	3.90×10^{8} M^{-1} s^{-1}	8
6	$HO^{\bullet} + HCO_3^{-} \rightarrow CO_3^{\bullet-} + H_2O$	8.50×10^{6} M^{-1} s^{-1}	8

7	$HO^{\bullet} + H_2CO_3 \rightarrow CO_3^{\bullet-} + H_2O + H^+$	1.00×10^{6} M^{-1} s^{-1}	8
8	$H_2O_2 + CO_3^{\bullet-} \rightarrow HCO_3^- + HO_2^{\bullet}$	4.30×10^{5} M^{-1} s^{-1}	8
9	$HO_2^- + CO_3^{\bullet-} \rightarrow HCO_3^- + O_2$	3.00×10^{7} M^{-1} s^{-1}	8
10	$HO^{\bullet} + CO_3^{\bullet-} \rightarrow$ products	3.00×10^{9} M^{-1} s^{-1}	8
11	$O_2^{\bullet-} + CO_3^{\bullet-} \rightarrow CO_3^{2-} + O_2$	6.00×10^{8} M^{-1} s^{-1}	8
12	$CO_3^{\bullet-} + CO_3^{\bullet-} \rightarrow$ products	3.00×10^{7} M^{-1} s^{-1}	8
	**Reactive Chlorine Species (w/ ROS, Bicarbonate) (72 reactions)**		
1	$H^+ + OCl^- \rightarrow HOCl$	5.00×10^{10} M^{-1} s^{-1}	8
2	$HOCl \rightarrow H^+ + OCl^-$	1.60×10^{3} s^{-1}	8
3	$H^+ + Cl^- \rightarrow HCl$	5.00×10^{10} M^{-1} s^{-1}	8
4	$HCl \rightarrow H^+ + Cl^-$	8.60×10^{16} s^{-1}	8
5	$HO^{\bullet} + Cl^- \rightarrow ClOH^{\bullet-}$	4.30×10^{9} M^{-1} s^{-1}	8
6	$ClOH^{\bullet-} \rightarrow HO^{\bullet} + Cl^-$	6.10×10^{9} s^{-1}	8
7	$ClOH^{\bullet-} + H^+ \rightarrow Cl^{\bullet} + H_2O$	2.10×10^{10} M^{-1} s^{-1}	8
8	$ClOH^{\bullet-} + Cl^- \rightarrow Cl_2^{\bullet-} + OH^-$	1.00×10^{5} M^{-1} s^{-1}	8
9	$Cl^{\bullet} + H_2O \rightarrow ClOH^{\bullet-} + H^+$	4.50×10^{3} M^{-1} s^{-1}	8
10	$Cl^{\bullet} + OH^- \rightarrow ClOH^{\bullet-}$	1.80×10^{10} M^{-1} s^{-1}	8
11	$Cl^{\bullet} + H_2O_2 \rightarrow HO_2^{\bullet} + Cl^- + H^+$	2.00×10^{9} M^{-1} s^{-1}	8
12	$Cl^{\bullet} + Cl^- \rightarrow Cl_2^{\bullet-}$	8.50×10^{9} M^{-1} s^{-1}	8
13	$Cl^{\bullet} + Cl^{\bullet} \rightarrow Cl_2$	8.80×10^{7} M^{-1} s^{-1}	8
14	$Cl^{\bullet} + HOCl \rightarrow ClO^{\bullet} + H^+ + Cl^-$	3.00×10^{9} M^{-1} s^{-1}	8
15	$Cl^{\bullet} + OCl^- \rightarrow ClO^{\bullet} + Cl^-$	8.30×10^{9} M^{-1} s^{-1}	8
16	$Cl_2^{\bullet-} \rightarrow Cl^{\bullet} + Cl^-$	6.00×10^{4} s^{-1}	8
17	$Cl_2 + OH^- \rightarrow HOCl + Cl^-$	1.00×10^{9} M^{-1} s^{-1}	8
18	$Cl_2^{\bullet-} + Cl_2^{\bullet-} \rightarrow Cl_2 + 2Cl^-$	8.30×10^{8} M^{-1} s^{-1}	8
19	$Cl_2^{\bullet-} + Cl^{\bullet} \rightarrow Cl_2 + Cl^-$	2.10×10^{9} M^{-1} s^{-1}	8
20	$Cl_2^{\bullet-} + H_2O_2 \rightarrow HO_2^{\bullet} + 2Cl^- + H^+$	1.40×10^{5} M^{-1} s^{-1}	8
21	$Cl_2^{\bullet-} + HO_2^{\bullet} \rightarrow O_2 + 2Cl^- + H^+$	3.00×10^{9} M^{-1} s^{-1}	8
22	$Cl_2^{\bullet-} + O_2^{\bullet-} \rightarrow O_2 + 2Cl^-$	1.00×10^{9} M^{-1} s^{-1}	8
23	$Cl_2^{\bullet-} + H_2O \rightarrow Cl^- + HClOH$	1.30×10^{3} M^{-1} s^{-1}	8
24	$Cl_2^{\bullet-} + OH^- \rightarrow Cl^- + ClOH^{\bullet-}$	4.50×10^{7} M^{-1} s^{-1}	8
25	$HClOH \rightarrow ClOH^{\bullet-} + H^+$	1.00×10^{8} s^{-1}	8
26	$HClOH \rightarrow Cl^{\bullet} + H_2O$	1.00×10^{2} s^{-1}	8
27	$HClOH + Cl^- \rightarrow Cl_2^{\bullet-} + H_2O$	5.00×10^{9} M^{-1} s^{-1}	8
28	$Cl_2 + Cl^- \rightarrow Cl_3^-$	2.00×10^{4} M^{-1} s^{-1}	8

29	$Cl_3^- \rightarrow Cl_2 + Cl^-$	1.10×10^5 s^{-1}	8
30	$Cl_3^- + HO_2^\bullet \rightarrow Cl_2^{\bullet-} + HCl + O_2$	1.00×10^9 M^{-1} s^{-1}	8
31	$Cl_3^- + O_2^{\bullet-} \rightarrow Cl_2^{\bullet-} + Cl^- + O_2$	3.80×10^9 M^{-1} s^{-1}	8
32	$Cl_2 + H_2O \rightarrow Cl^- + HOCl + H^+$	2.70×10^{-1} M^{-1} s^{-1}	8
33	$Cl^- + HOCl + H^+ \rightarrow Cl_2 + H_2O$	1.82×10^{-1} M^{-2} s^{-1}	8
34	$Cl_2 + H_2O_2 \rightarrow O_2 + 2HCl$	1.30×10^4 M^{-1} s^{-1}	8
35	$Cl_2 + O_2^{\bullet-} \rightarrow O_2 + Cl_2^{\bullet-}$	1.00×10^9 M^{-1} s^{-1}	8
36	$Cl_2 + HO_2^\bullet \rightarrow H^+ + O_2 + Cl_2^{\bullet-}$	1.00×10^9 M^{-1} s^{-1}	8
37	$HOCl + H_2O_2 \rightarrow HCl + H_2O + O_2$	1.10×10^4 M^{-1} s^{-1}	8
38	$OCl^- + H_2O_2 \rightarrow Cl^- + H_2O + O_2$	1.70×10^5 M^{-1} s^{-1}	8
39	$HOCl + HO^\bullet \rightarrow ClO^\bullet + H_2O$	2.00×10^9 M^{-1} s^{-1}	8
40	$HOCl + O_2^{\bullet-} \rightarrow Cl^\bullet + OH^- + O_2$	7.50×10^6 M^{-1} s^{-1}	8
41	$HOCl + HO_2^\bullet \rightarrow Cl^\bullet + H_2O + O_2$	7.50×10^6 M^{-1} s^{-1}	8
42	$OCl^- + HO^\bullet \rightarrow ClO^\bullet + OH^-$	8.80×10^9 M^{-1} s^{-1}	8
43	$OCl^- + O_2^{\bullet-} + H_2O \rightarrow Cl^\bullet + 2OH^- + O_2$	2.00×10^8 M^{-2} s^{-1}	8
44	$OCl^- + CO_3^{\bullet-} \rightarrow ClO^\bullet + CO_3^{2-}$	5.70×10^5 M^{-1} s^{-1}	8
45	$Cl^\bullet + CO_3^{2-} \rightarrow Cl^- + CO_3^{\bullet-}$	5.00×10^8 M^{-1} s^{-1}	8
46	$Cl^\bullet + HCO_3^- \rightarrow Cl^- + CO_3^{\bullet-} + H^+$	2.20×10^8 M^{-1} s^{-1}	8
47	$Cl_2^{\bullet-} + CO_3^{2-} \rightarrow 2Cl^- + CO_3^{\bullet-}$	1.60×10^8 M^{-1} s^{-1}	8
48	$ClO^\bullet + CO_3^{2-} \rightarrow OCl^- + CO_3^{\bullet-}$	6.00×10^2 M^{-1} s^{-1}	8
49	$Cl_2^{\bullet-} + HCO_3^- \rightarrow 2Cl^- + CO_3^{\bullet-} + H^+$	8.00×10^7 M^{-1} s^{-1}	8
50	$ClO^\bullet + ClO^\bullet \rightarrow Cl_2O_2$	2.50×10^9 M^{-1} s^{-1}	8
51	$2ClO^\bullet + H_2O \rightarrow HOCl + H^+ + ClO_2^-$	2.50×10^9 M^{-1} s^{-1}	8
52	$2ClO^\bullet + OH^- \rightarrow OCl^- + H^+ + ClO_2^-$	2.50×10^9 M^{-1} s^{-1}	8
53	$HO^\bullet + ClO^\bullet \rightarrow ClO_2^- + H^+$	1.00×10^9 M^{-1} s^{-1}	8
54	$HO^\bullet + ClO_2^- \rightarrow ClO_2^\bullet + OH^-$	6.30×10^9 M^{-1} s^{-1}	8
55	$HO^\bullet + ClO_3^- \rightarrow$ products	1.00×10^5 M^{-1} s^{-1}	8
56	$HO^\bullet + ClO_2^\bullet \rightarrow ClO_3^- + H^+$	4.00×10^9 M^{-1} s^{-1}	8
57	$Cl^\bullet + ClO_3^- \rightarrow$ products	1.00×10^5 M^{-1} s^{-1}	8
58	$Cl^\bullet + ClO_2^- \rightarrow ClO_2^\bullet + Cl^-$	7.00×10^9 M^{-1} s^{-1}	8
59	$Cl^\bullet + ClO_2^\bullet \rightarrow$ products	4.00×10^9 M^{-1} s^{-1}	8
60	$Cl_2^{\bullet-} + ClO_2^- \rightarrow ClO_2^\bullet + 2Cl^-$	1.30×10^8 M^{-1} s^{-1}	8
61	$ClO^\bullet + ClO_2^- \rightarrow OCl^- + ClO_2^\bullet$	9.40×10^8 M^{-1} s^{-1}	8
62	$Cl_2O_2 + H_2O \rightarrow ClO_2^- + HOCl + H^+$	1.00×10^4 s^{-1}	10
63	$Cl^\bullet + ClO_2^- \rightarrow ClO_2 + Cl^-$	7.00×10^9 $M^{-1}s^{-1}$	10

64	$Cl^{\bullet} + ClO_2 \rightarrow$ products	4.00×10^{9} $M^{-1}s^{-1}$	10
65	$HO^{\bullet} + ClO_3^{-} \rightarrow$ products	$<1.00\times10^{6}$ $M^{-1}s^{-1}$	10
66	$Cl^{\bullet} + ClO_3^{-} \rightarrow$ products	$<1.00\times10^{6}$ $M^{-1}s^{-1}$	10
67	$ClO_2^{\bullet} \rightarrow O_2 + Cl^{\bullet}$	6.70×10^{9} M^{-1} s^{-1}	9
68	$ClO_2^{\bullet} + HO^{\bullet} \rightarrow ClO_3^{-} + H^{+}$	4.00×10^{9} M^{-1} s^{-1}	9
69	$O^{\bullet-} + ClO_2 \rightarrow ClO_3^{-}$	2.70×10^{9} M^{-1} s^{-1}	9
70	$O_2^{\bullet-} + ClO_2^{-} \rightarrow$ products	4.00 M^{-1} s^{-1}	9
71	$O_2^{\bullet-} + ClO_3^{-} \rightarrow$ products	3.00×10^{-3} M^{-1} s^{-1}	9
72	$O^{\bullet-} + ClO_2^{-} \rightarrow OH^{-} + ClO_2^{\bullet}$	1.95×10^{8} M^{-1} s^{-1}	9
	**Reactive Nitrogen Species Produced by Oxidation of Chloramine (8 reactions)**		
1	$NH_2Cl + HO^{\bullet} \rightarrow {}^{\bullet}NHCl + H_2O$	1.02×10^{9} $M^{-1}s^{-1}$	10
2	$NH_2Cl + Cl^{\bullet} \rightarrow {}^{\bullet}NHCl + Cl^{-}$	1.00×10^{9} $M^{-1}s^{-1}$	10
3	$NH_2Cl + Cl_2^{\bullet-} \rightarrow {}^{\bullet}NHCl + 2Cl^{-} + H^{+}$	1.14×10^{7} $M^{-1}s^{-1}$	10
4	$NHCl_2 + HO^{\bullet} \rightarrow NCl_2^{\bullet} + H_2O$	6.21×10^{8} $M^{-1}s^{-1}$	10
5	$NHCl_2 + Cl^{\bullet} \rightarrow NCl_2^{\bullet} + Cl^{-} + H^{+}$	1.00×10^{9} $M^{-1}s^{-1}$	10
6	$NHCl_2 + Cl_2^{\bullet-} \rightarrow NCl_2^{\bullet} + 2Cl^{-} + H^{+}$	4.40×10^{6} $M^{-1}s^{-1}$	10
7	$NH_2Cl + {}^{\bullet}NH_2 \rightarrow {}^{\bullet}NHCl + NH_3$	1.00×10^{5} $M^{-1}s^{-1}$	10
8	$NHCl_2 + {}^{\bullet}NH_2 \rightarrow NCl_2^{\bullet} + NH_3$	1.00×10^{5} $M^{-1}s^{-1}$	10
	**Reactive Nitrogen Species Produced from Oxidation of Nitrite/Nitrate (8 reactions)**		
1	$HNO_2 \rightarrow NO_2^{-} + H^{+}$	2.51×10^{7} s^{-1}	11
2	$NO_2^{-} + H^{+} \rightarrow HNO_2$	2.10×10^{9} $M^{-1}s^{-1}$	11
3	$HNO_2 + HO^{\bullet} \rightarrow {}^{\bullet}NO_2 + H_2O$	2.60×10^{9} $M^{-1}s^{-1}$	11
4	$NO_2^{-} + HO^{\bullet} \rightarrow {}^{\bullet}NO_2 + OH^{-}$	1.20×10^{10} $M^{-1}s^{-1}$	11
5	$NO_2^{-} + CO_3^{\bullet-} \rightarrow {}^{\bullet}NO_2 + CO_3^{2-}$	6.60×10^{5} $M^{-1}s^{-1}$	11
6	$NO_2^{-} + Cl^{\bullet} \rightarrow {}^{\bullet}NO_2 + Cl^{-}$	5.00×10^{9} $M^{-1}s^{-1}$	12
7	$NO_3^{-} + HO^{\bullet} \rightarrow NO_3^{\bullet} + OH^{-}$	5.00×10^{8} $M^{-1}s^{-1}$	12
8	$NO_3^{-} + Cl^{\bullet} \rightarrow NO_3^{\bullet} + Cl^{-}$	1.00×10^{8} $M^{-1}s^{-1}$	12
	**Reactive Nitrogen Species Produced by Oxidation of Ammonia (8 reactions)**		
1	$NH_4^{+} \rightarrow NH_3 + H^{+}$	42.00 s^{-1}	12
2	$NH_3 + H^{+} \rightarrow NH_4^{+}$	8.40×10^{10} $M^{-1}s^{-1}$	12
3	$NH_4^{+} + HO^{\bullet} \rightarrow {}^{\bullet}NH_2 + H_2O + H^{+}$	1.38×10^{7} $M^{-1}s^{-1}$	13
4	$NH_3 + HO^{\bullet} \rightarrow {}^{\bullet}NH_2 + H_2O$	1.20×10^{8} $M^{-1}s^{-1}$	13
5	$NH_4^{+} + Cl^{\bullet} \rightarrow {}^{\bullet}NH_2 + 2H^{+} + 2Cl^{-}$	2.59×10^{8} $M^{-1}s^{-1}$	13
6	$NH_3 + Cl^{\bullet} \rightarrow {}^{\bullet}NH_2 + H^{+} + Cl^{-}$	4.30×10^{9} $M^{-1}s^{-1}$	13
7	$NH_4^{+} + Cl_2^{\bullet-} \rightarrow {}^{\bullet}NH_2 + 2Cl^{-} + 2H^{+}$	3.45×10^{5} $M^{-1}s^{-1}$	13

8	$NH_3 + Cl_2^{\bullet-} \rightarrow {}^{\bullet}NH_2 + H^+ + 2Cl^-$	5.70×10^6 $M^{-1}s^{-1}$	13
	**Speciation of Reactive Nitrogen Species (26 reactions)**		
1	${}^{\bullet}NH_2 + O_2 \rightarrow NH_2O_2^{\bullet}$	1.20×10^8 $M^{-1}s^{-1}$	10
2	${}^{\bullet}NHCl + O_2 \rightarrow NHClO_2^{\bullet}$	1.20×10^8 $M^{-1}s^{-1}$	10
3	$NH_2O_2^{\bullet} \rightarrow {}^{\bullet}NO + H_2O$	1.00×10^8 s^{-1}	10
4	$NHClO_2^{\bullet} \rightarrow {}^{\bullet}NO$ + products	1.00×10^8 s^{-1}	10
5	$NH_2O_2^{\bullet} \rightarrow$ transient species $\rightarrow N_2O$	5.98×10^8 s^{-1}	10
6	$NHClO_2^{\bullet} \rightarrow$ transient species $\rightarrow N_2O$	6.70×10^8 s^{-1}	10
7	${}^{\bullet}NO + HO^{\bullet} \rightarrow NO_2^- + H^+$	1.00×10^{10} $M^{-1}s^{-1}$	10
8	${}^{\bullet}NO + {}^{\bullet}NO + O_2 \rightarrow 2\ {}^{\bullet}NO_2$	2.10×10^6 $M^{-2}s^{-1}$	10
9	${}^{\bullet}NO + {}^{\bullet}NO_2 \rightarrow N_2O_3$	1.10×10^9 $M^{-1}s^{-1}$	10
10	$N_2O_3 \rightarrow {}^{\bullet}NO + {}^{\bullet}NO_2$	4.30×10^6 s^{-1}	10
11	$N_2O_3 + H_2O \rightarrow 2\ NO_2^- + 2H^+$	1.60×10^3 s^{-1}	10
12	${}^{\bullet}NO_2 + {}^{\bullet}NO_2 \rightarrow N_2O_4$	4.50×10^8 $M^{-1}s^{-1}$	10
13	$N_2O_4 + H_2O \rightarrow NO_2^- + NO_3^- + 2H^+$	1.00×10^3 s^{-1}	10
14	${}^{\bullet}NO_2 + {}^{\bullet}NO_2 \rightarrow N_2O_4$	4.5×10^8 $M^{-1}s^{-1}$	11
15	$N_2O_4 \rightarrow {}^{\bullet}NO_2 + {}^{\bullet}NO_2$	7.00×10^3 s^{-1}	11
16	${}^{\bullet}NO_2 + {}^{\bullet}NO_2 + H_2O \rightarrow NO_2^- + NO_3^- + 2H^+$	2.00×10^8 $M^{-2}s^{-1}$	11
17	${}^{\bullet}NO_2 + HO^{\bullet} \rightarrow HO_2NO$	4.50×10^9 $M^{-1}s^{-1}$	11
18	${}^{\bullet}NO_2 + HO_2^{\bullet} \rightarrow HO_2NO_2$	1.80×10^9 $M^{-1}s^{-1}$	11
19	${}^{\bullet}NO_2 + O_2^{\bullet-} \rightarrow O_2NO_2^-$	4.50×10^9 $M^{-1}s^{-1}$	11
20	$O_2NO_2^- \rightarrow {}^{\bullet}NO_2 + O_2^{\bullet-}$	1.00 s^{-1}	11
21	$O_2NO_2^- \rightarrow NO_2^- + O_2$	1.00 s^{-1}	11
22	${}^{\bullet}NO_2 + CO_3^{\bullet-} \rightarrow NO_3^- + CO2$	1.00×10^9 $M^{-1}s^{-1}$	11
23	$NO_3^{\bullet} + H_2O \rightarrow HNO_3 + HO^{\bullet}$	2.90×10^7 $M^{-1}s^{-1}$	11
24	$NO_3^{\bullet} + Br^- \rightarrow NO_3^- + Br^{\bullet}$	4.00×10^9 $M^{-1}s^{-1}$	14
25	$NO_3^{\bullet} + Cl^- \rightarrow NO_3^- + Cl^{\bullet}$	7.10×10^7 $M^{-1}s^{-1}$	14
26	$NO_3^{\bullet} + NO_3^{\bullet} \rightarrow N_2O_6$	7.90×10^5 $M^{-1}s^{-1}$	14
	**Peroxynitrite (24 reactions)**		
1	${}^{\bullet}NO + O_2^{\bullet-} \rightarrow ONOO^-$	6.70×10^9 $M^{-1}s^{-1}$	15
2	$ONOO^- \rightarrow {}^{\bullet}NO + O_2^{\bullet-}$	2.00×10^{-2} s^{-1}	15
3	$ONOO^- + CO_2 \rightarrow {}^{\bullet}NO_2 + CO_3^{\bullet-}$	2.90×10^4 $M^{-1}s^{-1}$	15
4	$ONOO^- \rightarrow NO_3^-$	8.00×10^{-6} s^{-1}	15
5	$ONOO^- \rightarrow {}^{\bullet}NO_2 + O^{\bullet-}$	1.00×10^{-6} s^{-1}	15
6	${}^{\bullet}NO_2 + O^{\bullet-} \rightarrow ONOO^-$	3.50×10^9 $M^{-1}s^{-1}$	15

7	$ONOO^- + {}^{\bullet}OH \rightarrow {}^{\bullet}NO + O_2 + OH^-$	4.80×10^9 $M^{-1}s^{-1}$	15
8	$ONOO^- + CO_3^{\bullet-} \rightarrow {}^{\bullet}NO + O_2 + CO_3^{2-}$	3.70×10^6 $M^{-1}s^{-1}$	15
9	${}^{\bullet}NO_2 + {}^{\bullet}OH \rightarrow ONOOH$	4.50×10^9 $M^{-1}s^{-1}$	15
10	$ONOOH \rightarrow {}^{\bullet}NO_2 + {}^{\bullet}OH$	3.50×10^{-1} s^{-1}	15
11	${}^{\bullet}NO + HO_2^{\bullet} \rightarrow ONOOH$	3.20×10^9 $M^{-1}s^{-1}$	15
12	$ONOOH \rightarrow NO_3^- + H+$	9.00×10^{-1} s^{-1}	15
13	$ONOOH + H^+ \rightarrow NO_3^- + 2H^+$	4.30 $M^{-1}s^{-1}$	15
14	$ONOOH + H_2O + H^+ \rightarrow HNO_2 + H_2O_2 + H^+$	1.10×10^{-1} $M^{-2}s^{-1}$	15
15	$HNO_2 + H_2O_2 + H^+ \rightarrow ONOOH + H_2O + H^+$	9.60×10^3 $M^{-2}s^{-1}$	15
16	${}^{\bullet}NO_2 + O_2^{\bullet-} \rightarrow O_2NOO^-$	4.50×10^9 $M^{-1}s^{-1}$	15
17	$O_2NOO^- \rightarrow {}^{\bullet}NO_2 + O_2^{\bullet-}$	1.00 s^{-1}	15
18	$O_2NOO^- \rightarrow NO_2^- + O_2$	1.40 s^{-1}	15
19	${}^{\bullet}NO_2 + HO_2^{\bullet} \rightarrow O_2NOOH$	1.80×10^9 $M^{-1}s^{-1}$	15
20	$O_2NOOH \rightarrow {}^{\bullet}NO_2 + HO_2^{\bullet}$	2.60×10^{-2} s^{-1}	15
21	$H^+ + ONOO^- \rightarrow ONOOH$	5.00×10^{10} $M^{-1}s^{-1}$	15
22	$ONOOH \rightarrow H^+ + ONOO^-$	1.25×10^4 s^{-1}	15
23	$H^+ + O_2NOO^- \rightarrow O_2NOOH$	5.00×10^{10} $M^{-1}s^{-1}$	15
24	$O_2NOOH \rightarrow H^+ + O_2NOO^-$	6.90×10^4 s^{-1}	15
	**Breakpoint Chlorination (16 reactions)**		
1	$HOCl + NH_3 \rightarrow NH_2Cl + H_2O$	4.17×10^6 $M^{-1}s^{-1}$	10
2	$NH_2Cl + H_2O \rightarrow HOCl + NH_3$	2.11×10^{-5} s^{-1}	10
3	$NH_2Cl + HOCl \rightarrow NHCl_2 + H_2O$	2.78×10^2 $M^{-1}s^{-1}$	10
4	$NHCl_2 + H_2O \rightarrow NH_2Cl + HOCl$	6.39×10^{-7} s^{-1}	10
5	$NH_2Cl + NH_2Cl \rightarrow NHCl_2 + NH_3$	1.10×10^{-2} M^{-1} s^{-1}	10
6	$NHCl_2 + NH_3 \rightarrow NH_2Cl + NH_2Cl$	9.51×10^{-2} M^{-1} s^{-1}	10
7	$NHCl_2 + H_2O \rightarrow$ intermediate	6.94×10^{-7} s^{-1}	10
8	$NHCl_2$ + intermediate $\rightarrow$ HOCl + products	2.78×10^4 M^{-1} s^{-1}	10
9	NH_2Cl + intermediate $\rightarrow$ products	8.30×10^3 M^{-1} s^{-1}	10
10	$NH_2Cl + NHCl_2 \rightarrow$ products	1.53×10^{-2} M^{-1} s^{-1}	10
11	$HOCl + NHCl_2 \rightarrow NCl_3 + H_2O$	20.80 M^{-1} s^{-1}	10
12	$NCl_3 + H_2O \rightarrow HOCl + NHCl_2$	3.20×10^{-5} s^{-1}	10
13	$HOCl + NHCl_2 \rightarrow NCl_3 + H_2O$	20.80 M^{-1} s^{-1}	10
14	$NCl_3 + NHCl_2 + 2H_2O \rightarrow 2HOCl$ + products	3.51×10^2 M^{-1} s^{-1}	10
15	$NCl_3 + NH_2Cl + H_2O \rightarrow HOCl$ + products	8.76 M^{-1} s^{-1}	10
16	$NHCl_2 + 2\ OCl^- + H_2O \rightarrow NO_3^- + 3H^+ + 4Cl^-$	2.31×10^2 M^{-1} s^{-1}	10

	**Organic Radicals (36 reactions)**		
1	$CH_3C(O)OH + HO^{\bullet} \rightarrow {}^{\bullet}CH_2C(O)OH + H_2O$	1.60×10^{7} M^{-1} s^{-1}	16
2	$CH_3C(O)O^{-} + HO^{\bullet} \rightarrow {}^{\bullet}CH_2C(O)O^{-} + H_2O$	8.50×10^{7} M^{-1} s^{-1}	16
3	$CH_2C(O)O^{-} + O_2 \rightarrow {}^{\bullet}OOCH_2C(O)O^{-}$	1.70×10^{9} M^{-1} s^{-1}	16
4	$2{}^{\bullet}OOCH_2C(O)O^{-} \rightarrow HOCC(O)O^{-} + HOCH_2C(O)O^{-} + O_2$	2.20×10^{7} M^{-1} s^{-1}	16
5	$2\ {}^{\bullet}OOCH_2C(O)O^{-} \rightarrow 2\ HOCC(O)O^{-} + H_2O_2$	2.30×10^{7} M^{-1} s^{-1}	16
6	$2\ {}^{\bullet}OOCH_2C(O)O^{-} \rightarrow 2\ {}^{\bullet}OCH_2C(O)O^{-} + O_2$	3.00×10^{7} M^{-1} s^{-1}	16
7	${}^{\bullet}OOCH_2C(O)O^{-} + HO_2^{\bullet} \rightarrow HOCH_2C(O)O^{-} + {}^{3}O + {}^{3}O_2$	9.80×10^{6} M^{-1} s^{-1}	16
8	$2{}^{\bullet}OCH_2C(O)O^{-} \rightarrow HOCH_2C(O)O^{-} + HOCC(O)O^{-}$	7.30×10^{9} M^{-1} s^{-1}	16
9	${}^{\bullet}OCH_2C(O)O^{-} \rightarrow HCHO + {}^{\bullet}C(O)O^{-}$	1.00×10^{6} s^{-1}	16
10	${}^{\bullet}C(O)O^{-} + O_2 \rightarrow CO_2 + {}^{\bullet}O_2^{-}$	2.40×10^{9} M^{-1} s^{-1}	16
11	$HOCC(O)O^{-} + HO^{\bullet} \rightarrow {}^{\bullet}OCC(O)O^{-} + H_2O$	2.60×10^{9} M^{-1} s^{-1}	16
12	$HOCH_2C(O)O^{-} + HO^{\bullet} \rightarrow HO{}^{\bullet}CHC(O)O^{-} + H_2O$	8.60×10^{8} M^{-1} s^{-1}	16
14	$HCHO + HO^{\bullet} \rightarrow$ products	1.00×10^{9} M^{-1} s^{-1}	16
15	$CH_3C(O)OOH + HO^{\bullet} \rightarrow CH_3C(O)OO^{\bullet} + H_2O$	1.30×10^{9} M^{-1} s^{-1}	16
16	$CH_3C(O)OOH + CH_3C(O)O^{\bullet} \rightarrow CH_3C(O)OO^{\bullet} + CH_3C(O)O^{-}$	1.00×10^{7} M^{-1} s^{-1}	16
17	$CH_3C(O)OOH + HO_2^{\bullet} \rightarrow CH_3C(O)OO^{\bullet} + H_2O_2$	2.00×10^{2} M^{-1} s^{-1}	16
18	$CH_3C(O)OO^{\bullet} + CH_3C(O)OO^{\bullet} \rightarrow 2\ CH_3C(O)O^{\bullet} + O_2$	8.30×10^{9} M^{-1} s^{-1}	16
19	$CH_3C(O)OO^{\bullet} + HO_2^{\bullet} \rightarrow CH_3C(O)OH + {}^{3}O + {}^{3}O_2$	2.00×10^{6} M^{-1} s^{-1}	16
20	$CH_3C(O)OO^{\bullet} \rightarrow HO_2^{\bullet} + CH_2CO$	1.82 s^{-1}	16
21	$CH_2CO + H_2O \rightarrow CH_3C(O)OH$	44.00 s^{-1}	16
22	$CH_3C(O)O^{\bullet} + CH_3C(O)O^{\bullet} \rightarrow (CH_3C(O)O)_2$	1.00×10^{9} M^{-1} s^{-1}	16
23	$CH_3C(O)O^{\bullet} \rightarrow {}^{\bullet}CH_3 + CO_2$	2.30×10^{5} s^{-1}	16
24	${}^{\bullet}CH_3 + O_2 \rightarrow {}^{\bullet}OOCH_3$	4.70×10^{9} M^{-1} s^{-1}	16
25	${}^{\bullet}OOCH_3 + {}^{\bullet}OOCH_3 \rightarrow HCHO + CH_3OH + O_2$	2.80×10^{8} M^{-1} s^{-1}	16
26	${}^{\bullet}OOCH_3 + {}^{\bullet}OOCH_3 \rightarrow 2\ HCHO + H_2O_2$	2.50×10^{7} M^{-1} s^{-1}	16
27	${}^{\bullet}OOCH_3 + {}^{\bullet}OOCH_3 \rightarrow 2\ {}^{\bullet}OCH_3 + O_2$	2.20×10^{8} M^{-1} s^{-1}	16
28	${}^{\bullet}OOCH_3 + HO_2^{\bullet} \rightarrow CH_3OH + {}^{3}O + {}^{3}O_2$	5.00×10^{8} M^{-1} s^{-1}	16
29	${}^{\bullet}OCH_3 \rightarrow {}^{\bullet}CH_2OH$	5.00×10^{5} s^{-1}	16
30	${}^{\bullet}CH_2OH + O_2 \rightarrow OHCH_2OO^{\bullet}$	4.90×10^{9} M^{-1} s^{-1}	16
31	$OHCH_2OO^{\bullet} \rightarrow HCHO + HO_2^{\bullet}$	10.00 s^{-1}	16
32	$OHCH_2OO^{\bullet} + OH^{-} \rightarrow HCHO + O_2^{\bullet-} + H_2O$	1.50×10^{10} M^{-1} s^{-1}	16
33	$OHCH_2OO^{\bullet} + OHCH_2OO^{\bullet} \rightarrow 2\ OHCH_2O^{\bullet} + O_2$	8.90×10^{8} M^{-1} s^{-1}	16
34	$OHCH_2OO^{\bullet} + OHCH_2OO^{\bullet} \rightarrow 2\ HCOOH + H_2O_2$	1.60×10^{9} M^{-1} s^{-1}	16
35	$OHCH_2OO^{\bullet} + HO_2^{\bullet} \rightarrow CH_2(OH)_2 + {}^{3}O + O_2$	2.00×10^{6} M^{-1} s^{-1}	16

36	$CH_3OH + HO^\bullet \rightarrow {}^\bullet CH_2OH$ (93%) + ${}^\bullet OCH_3$ (7%) + H_2O	9.70×10^{8} M^{-1} s^{-1}	16
	**Reactive Bromine Species (w/ ROS, Bicarbonate) (61 reactions)**		
1	$H^+ + Br^- \rightarrow HBr$	5.00×10^{10} M^{-1} s^{-1}	17
2	$HBr \rightarrow H^+ + Br^-$	5.00×10^{19} s^{-1}	17
3	$H^+ + OBr^- \rightarrow HOBr$	5.00×10^{10} M^{-1} s^{-1}	17
4	$HOBr \rightarrow H^+ + OBr$-	7.90×10^{1} s^{-1}	17
5	$HO^\bullet + Br^- \rightarrow BrOH^{\bullet-}$	1.10×10^{10} M^{-1} s^{-1}	17
6	$BrOH^{\bullet-} + Br^- \rightarrow Br_2^{\bullet-} + OH^-$	1.90×10^{8} M^{-1} s^{-1}	17
7	$Br^\bullet + Br^- \rightarrow Br_2^{\bullet-}$	1.20×10^{10} M^{-1} s^{-1}	17
8	$Br_2 + Br$- $\rightarrow Br_3^-$	9.60×10^{8} M^{-1} s^{-1}	17
9	$HOBr + Br^- \rightarrow Br_2OH^-$	5.00×10^{9} M^{-1} s^{-1}	17
10	$CO_3^{\bullet-} + Br^- \rightarrow Br^\bullet + CO_3^{2-}$	1.00×10^{5} M^{-1} s^{-1}	17
11	$BrOH^{\bullet-} \rightarrow Br^- + HO^\bullet$	3.30×10^{7} s^{-1}	17
12	$BrOH^{\bullet-} \rightarrow Br^\bullet + OH^-$	4.20×10^{6} s^{-1}	17
13	$BrOH^{\bullet-} + H^+ \rightarrow Br^\bullet + H_2O$	4.40×10^{10} M^{-1} s^{-1}	17
14	$Br^\bullet + H_2O_2 \rightarrow HBr + HO_2^\bullet$	4.00×10^{9} M^{-1} s^{-1}	17
15	$Br^\bullet + OH^- \rightarrow BrOH^{\bullet-}$	1.30×10^{10} M^{-1} s^{-1}	17
16	$Br^\bullet + H_2O \rightarrow BrOH^{\bullet-} + H^+$	1.36 s^{-1}	17
17	$Br^\bullet + Br^\bullet \rightarrow Br_2$	1.00×10^{9} M^{-1} s^{-1}	17
18	$Br^\bullet + HO_2^\bullet \rightarrow HBr + O_2$	1.60×10^{8} M^{-1} s^{-1}	17
19	$Br^\bullet + CO_3^{2--} \rightarrow CO_3^{\bullet-} + Br$	2.00×10^{6} M^{-1} s^{-1}	17
20	$Br^\bullet + HCO_3^- \rightarrow HBr + CO_3^{\bullet-}$	1.00×10^{6} M^{-1} s^{-1}	17
21	$Br_2^{\bullet-} + HO_2^\bullet \rightarrow Br_2 + HO_2^-$	4.40×10^{9} M^{-1} s^{-1}	17
22	$Br_2^{\bullet-} + HO^\bullet \rightarrow HOBr + Br^-$	1.00×10^{9} M^{-1} s^{-1}	17
23	$Br_2^{\bullet-} + O_2^{\bullet-} \rightarrow 2\,Br^- + O_2$	1.70×10^{8} M^{-1} s^{-1}	17
24	$Br_2^{\bullet-} + OH^- \rightarrow BrOH^{\bullet-} + Br^-$	2.70×10^{6} M^{-1} s^{-1}	17
25	$Br_2^{\bullet-} \rightarrow Br^\bullet + Br^-$	1.90×10^{4} s^{-1}	17
26	$Br_2^{\bullet-} + Br_2^{\bullet-} \rightarrow Br_3^- + Br^-$	1.90×10^{9} M^{-1} s^{-1}	17
27	$Br_2^{\bullet-} + Br^\bullet \rightarrow Br_3^-$	2.00×10^{9} M^{-1} s^{-1}	17
28	$Br_2^{\bullet-} + Br^- \rightarrow$ products	9.05×10^{5} M^{-1} s^{-1}	17
29	$Br_2^{\bullet-} + OBr^- \rightarrow BrO^\bullet + 2Br^-$	8.00×10^{7} M^{-1} s^{-1}	17
30	$Br_2^{\bullet-} + H_2O_2 \rightarrow 2\,Br^- + HO_2^\bullet + H^+$	5.00×10^{2} M^{-1} s^{-1}	17
31	$Br_2^{\bullet-} + CO_3^{2-} \rightarrow CO_3^{\bullet-} + 2\,Br^-$	1.10×10^{5} M^{-1} s^{-1}	17
32	$Br_2^{\bullet-} + HCO_3^- \rightarrow CO_3^{\bullet-} + 2\,Br^- + H^+$	8.00×10^{4} M^{-1} s^{-1}	17
33	$Br_3^- + HO_2^\bullet \rightarrow Br_2^{\bullet-} + HBr + O2$	1.00×10^{7} M^{-1} s^{-1}	17

34	$Br_3^- + O_2^{\bullet-} \rightarrow Br_2^{\bullet-} + Br^- + O2$	3.80×10^9 M^{-1} s^{-1}	17
35	$Br_3^- \rightarrow Br_2 + Br^-$	8.30×10^8 s^{-1}	17
36	$Br_2 + O_2^{\bullet-} \rightarrow Br_2^{\bullet-} + O_2$	5.60×10^9 M^{-1} s^{-1}	17
37	$Br_2 + HO_2^{\bullet} \rightarrow Br_2^{\bullet-} + H^+ + O_2$	1.10×10^8 M^{-1} s^{-1}	17
38	$Br_2 + H_2O \rightarrow Br_2OH^- + H^+$	9.70×10^1 s^{-1}	17
39	$Br_2 + H_2O_2 \rightarrow 2\ HBr + O_2$	1.30×10^3 M^{-1} s^{-1}	17
40	$Br_2OH^- + H^+ \rightarrow Br_2 + H_2O$	2.00×10^{10} M^{-1} s^{-1}	17
41	$Br_2OH^- \rightarrow HOBr + Br^-$	5.00×10^9 s^{-1}	**17**
42	$HOBr + HO^{\bullet} \rightarrow BrO^{\bullet} + H_2O$	2.00×10^9 M^{-1} s^{-1}	**17**
43	$HOBr + Br^{\bullet} \rightarrow BrO^{\bullet} + HBr$	1.00×10^8 M^{-1} s^{-1}	17
44	$HOBr + O_2^{\bullet-} \rightarrow BrOH^{\bullet-} + O_2$	3.50×10^9 M^{-1} s^{-1}	17
45	$HOBr + HO_2^{\bullet} \rightarrow BrOH^{\bullet-} + H^+ + O_2$	3.50×10^9 M^{-1} s^{-1}	17
46	$HOBr + H_2O_2 \rightarrow HBr + H_2O + O_2$	3.50×10^4 M^{-1} s^{-1}	17
47	$HOBr + HO_2^- \rightarrow HBr + OH^- + O_2$	7.60×10^8 M^{-1} s^{-1}	17
48	$OBr^- + HO^{\bullet} \rightarrow BrO^{\bullet} + OH^-$	4.20×10^9 M^{-1} s^{-1}	17
49	$OBr^- + Br^{\bullet} \rightarrow BrO^{\bullet} + Br^-$	4.10×10^9 M^{-1} s^{-1}	17
50	$OBr^- + O_2^{\bullet-} + H_2O \rightarrow Br^{\bullet} + 2OH^- + O_2$	2.00×10^8 M^{-1} s^{-1}	17
51	$BrO^{\bullet} + BrO^{\bullet} \rightarrow BrO_2^- + Br^-$	2.80×10^9 M^{-1} s^{-1}	17
52	$2\ BrO^{\bullet} + H_2O \rightarrow OBr^- + BrO_2^- + 2H^+$	9.09×10^7 M^{-2} s^{-1}	17
53	$BrO^{\bullet} + BrO_2^- \rightarrow OBr^- + BrO_2$	3.40×10^8 M^{-1} s^{-1}	17
54	$Br_2^{\bullet-} + BrO_2^- \rightarrow BrO_2 + 2Br^-$	8.00×10^7 M^{-1} s^{-1}	17
55	$HO^{\bullet} + BrO_2^- \rightarrow BrO_2 + OH^-$	1.90×10^9 M^{-1} s^{-1}	17
56	$HO^{\bullet} + BrO_2 \rightarrow BrO_3^- + H^+$	2.00×10^9 M^{-1} s^{-1}	17
57	$BrO_2 + BrO_2 \rightarrow Br_2O_4$	1.40×10^9 M^{-1} s^{-1}	17
58	$Br_2O_4 \rightarrow 2\ BrO_2$	7.00×10^7 s^{-1}	17
59	$Br_2O_4 + OH^- \rightarrow BrO_3^- + BrO_2^- + H^+$	1.00×10^{10} M^{-1} s^{-1}	17
60	$OBr^- + H_2O_2 \rightarrow Br^- + H_2O + O_2$	1.20×10^6 M^{-1} s^{-1}	17
61	$OBr^- + CO_3^{\bullet-} \rightarrow CO_3^{2-} + BrO^{\bullet}$	4.3×10^7 M^{-1} s^{-1}	17
	**Reactive Bromine Species w/ Reactive Chlorine Species (39 reactions)**		
1	$ClOH^{\bullet-} + Br^- \rightarrow OH^- + BrCl^{\bullet-}$	1.00×10^9 M^{-1} s^{-1}	17
2	$Cl^{\bullet} + Br^- \rightarrow BrCl^{\bullet-}$	1.20×10^{10} M^{-1} s^{-1}	17
3	$Cl_2^{\bullet-} + Br^- \rightarrow BrCl^{\bullet-} + Cl^-$	4.00×10^9 M^{-1} s^{-1}	17
4	$BrOH^{\bullet-} + Cl^- \rightarrow BrCl^{\bullet-} + OH^-$	1.90×10^8 M^{-1} s^{-1}	17
5	$Br^{\bullet} + Cl^- \rightarrow BrCl^{\bullet-}$	1.00×10^8 M^{-1} s^{-1}	17
6	$Br_2^{\bullet-} + Cl^- \rightarrow BrCl^{\bullet-} + Br^-$	4.30×10^6 M^{-1} s^{-1}	17

7	$Br_2^{\bullet-} + Cl_2^{\bullet-} \rightarrow Br_2 + 2Cl^-$	4.00×10^9 M^{-1} s^{-1}	17
8	$BrCl^{\bullet-} + HO^{\bullet} \rightarrow BrCl + OH^-$	1.00×10^9 M^{-1} s^{-1}	17
9	$BrCl^{\bullet-} + HO_2^{\bullet} \rightarrow Br^- + Cl^- + O_2 + H^+$	1.00×10^9 M^{-1} s^{-1}	17
10	$BrCl^{\bullet-} + O_2^{\bullet-} \rightarrow Br^- + Cl^- + O_2$	6.00×10^8 M^{-1} s^{-1}	17
11	$BrCl^{\bullet-} + H_2O_2 \rightarrow HO_2^{\bullet} + Br^- + HCl$	5.00×10^3 M^{-1} s^{-1}	17
12	$BrCl^{\bullet-} + OH^- \rightarrow Br^- + ClOH^{\bullet-}$	3.00×10^6 M^{-1} s^{-1}	17
13	$BrCl^{\bullet-} + OH^- \rightarrow Cl^- + BrOH^{\bullet-}$	2.00×10^7 M^{-1} s^{-1}	17
14	$BrCl^{\bullet-} + HCO_3^- \rightarrow HCl + Br^- + CO_3^{\bullet-}$	3.00×10^6 M^{-1} s^{-1}	17
15	$BrCl^{\bullet-} + CO_3^{2-} \rightarrow Cl^- + Br^- + CO_3^{\bullet-}$	6.00×10^6 M^{-1} s^{-1}	17
16	$BrCl^{\bullet-} + BrCl^{\bullet-} \rightarrow BrCl + Br^- + Cl^-$	4.70×10^9 M^{-1} s^{-1}	17
17	$BrCl^{\bullet-} + Cl_2^{\bullet-} \rightarrow BrCl + 2Cl^-$	2.00×10^9 M^{-1} s^{-1}	17
18	$BrCl^{\bullet-} + Br_2^{\bullet-} \rightarrow Br2 + Cl^- + Br^-$	4.00×10^9 M^{-1} s^{-1}	17
19	$BrCl^{\bullet-} \rightarrow Cl^{\bullet} + Br^-$	1.90×10^3 s^{-1}	17
20	$BrCl^{\bullet-} \rightarrow Br^{\bullet} + Cl^-$	6.10×10^4 s^{-1}	17
21	$BrCl^{\bullet-} + Br^- \rightarrow Br_2^{\bullet-} + Cl^-$	8.00×10^9 M^{-1} s^{-1}	17
22	$BrCl^{\bullet-} + Cl^- \rightarrow Cl_2^{\bullet-} + Br^-$	1.10×10^2 M^{-1} s^{-1}	17
23	$Cl^- + HOBr \rightarrow BrCl + OH^-$	5.60×10^2 M^{-1} s^{-1}	17
24	$Br^- + HOCl \rightarrow BrCl + OH^-$	1.30×10^{-1} M^{-1} s^{-1}	17
25	$Br^- + HOCl \rightarrow HOBr + Cl^-$	6.84×10^3 M^{-1} s^{-1}	17
26	$Br^- + OCl^- \rightarrow OBr^- + Cl^-$	9.00×10^{-4} M^{-1} s^{-1}	17
27	$BrCl + H_2O \rightarrow HOBr + Cl^- + H^+$	1.00×10^5 s^{-1}	17
28	$BrCl + H_2O_2 \rightarrow Br^- + Cl^- + H_2O$	1.30×10^4 M^{-1} s^{-1}	17
29	$BrCl + O_2^{\bullet-} \rightarrow BrCl^{\bullet-} + O_2$	4.00×10^9 M^{-1} s^{-1}	17
30	$BrCl + HO_2^{\bullet} \rightarrow BrCl^{\bullet-} + O_2 + H^+$	5.00×10^8 M^{-1} s^{-1}	17
31	$BrCl + Cl^- \rightarrow BrCl_2^-$	1.00×10^6 M^{-1} s^{-1}	17
32	$BrCl_2^- \rightarrow BrCl + Cl^-$	1.70×10^5 s^{-1}	17
33	$BrCl + Br^- \rightarrow Br2Cl^-$	3.00×10^8 M^{-1} s^{-1}	17
34	$Br_2Cl^- \rightarrow BrCl + Br^-$	1.70×10^4 s^{-1}	17
35	$Br_2 + Cl^- \rightarrow Br_2Cl^-$	5.00×10^4 M^{-1} s^{-1}	17
36	$Cl_2 + Br^- \rightarrow BrCl_2^-$	6.00×10^9 M^{-1} s^{-1}	17
37	$BrCl_2^- \rightarrow Cl_2 + Br^-$	9.00×10^3 s^{-1}	17
38	$Br_2Cl^- + Cl^- \rightarrow BrCl_2^- + Br^-$	1.00×10^5 M^{-1} s^{-1}	17
39	$BrCl_2^- + Br^- \rightarrow Br_2Cl^- + Cl^-$	3.00×10^8 M^{-1} s^{-1}	17
	**Bromination (7 reactions)**		
1	$HOBr + NH_3 \rightarrow NH_2Br + H_2O$	5.10×10^6 M^{-1} s^{-1}	18

2	$NH_2Br + H_2O \rightarrow HOBr + NH_3$	1.50×10^{-3} s^{-1}	18
3	$HOBr + NH_2Cl \rightarrow NHBrCl + H_2O$	2.70×10^{5} M^{-1} s^{-1}	18
4	$NH_2Cl + Br^{-} \rightarrow NH_2Br + Cl^{-}$	1.40×10^{-2} M^{-1} s^{-1}	18
5	$NH_2Cl + Br^{-} \rightarrow int + Cl^{-}$	8.85×10^{-2} M^{-1} s^{-1}	18
6	$NH_2Cl + int \rightarrow NHBrCl + NH_3$	1.00×10^{12} M^{-1} s^{-1}	18
7	$NH_2Cl + NHBrCl \rightarrow N_2 + Br^{-} + 3H^{+} + 2Cl^{-}$	5.00×10^{-3} $M^{-1}s^{-1}$	18
	**Phosphate Buffer (15 reactions)**		
1	$H_2PO_4^{-} + H^{+} \rightarrow H_3PO_4$	5.00×10^{10} M^{-1} s^{-1}	8
2	$H_3PO_4 \rightarrow H_2PO_4^{-} + H^{+}$	3.87×10^{8} s^{-1}	8
3	$HPO_4^{2-} + H^{+} \rightarrow H_2PO_4^{-}$	5.00×10^{10} M^{-1} s^{-1}	8
4	$H_2PO_4^{-} \rightarrow HPO_4 + H^{+}$	3.15×10^{3} s^{-1}	8
5	$PO4^{3-} + H^{+} \rightarrow HPO_4^{2-}$	5.00×10^{10} M^{-1} s^{-1}	8
6	$HPO_4^{2-} \rightarrow PO_4^{3-} + H^{+}$	2.50×10^{-2} s^{-1}	8
7	$HO^{\bullet} + HPO_4^{2-} \rightarrow HPO_4^{\bullet-} + OH^{-}$	1.50×10^{5} M^{-1} s^{-1}	8
8	$HO^{\bullet} + H_2PO_4^{-} \rightarrow HPO_4^{\bullet-} + H_2O$	2.00×10^{4} M^{-1} s^{-1}	8
9	$H_2O_2 + HPO_4^{\bullet-} \rightarrow H_2PO_4^{-} + HO_2^{\bullet}$	2.70×10^{7} M^{-1} s^{-1}	8
10	$HPO_4^{\bullet-} + Cl^{-} \rightarrow Cl^{\bullet} + HPO_4^{2-}$	1.00×10^{4} $M^{-1}s^{-1}$	12
11	$HO^{\bullet} + H_3PO_4 \rightarrow H_2PO_4^{\bullet} + H_2O$	1.37×10^{6} $M^{-1}s^{-1}$	9
12	$H_2O_2 + H_2PO_4^{\bullet} + \rightarrow H_2PO_4^{-} + 2\ H^{+} + O_2^{\bullet-}$	5.50×10^{7} $M^{-1}s^{-1}$	9
13	$O^{\bullet-} + HPO_4^{2-} \rightarrow$ products	3.50×10^{6} $M^{-1}s^{-1}$	8
14	$H_2O + H_2PO_4^{\bullet} \rightarrow H_3PO_4 + HO^{\bullet}$	1.30×10^{5} $M^{-1}s^{-1}$	8
15	$Cl^{-} + H_2PO_4^{\bullet} \rightarrow H_2PO_4^{-} + Cl^{\bullet}$	2.20×10^{6} $M^{-1}s^{-1}$	8
	**Natural Organic Matter (NOM) (9 reactions)**		
1	$NOM + HO^{\bullet} \rightarrow$ products	2.95×10^{8} Mc^{-1} s^{-1}	19
2	$NOM + Cl^{\bullet} \rightarrow$ products	9.45×10^{8} Mc^{-1} s^{-1}	19
3	$NOM + Cl_2^{\bullet-} \rightarrow$ products	2.01×10^{7} Mc^{-1} s^{-1}	19
4	$NOM + ClO^{\bullet} \rightarrow$ products	5.4×10^{8} Mc^{-1} s^{-1}	17
5	$NOM + Br^{\bullet} \rightarrow$ products	2.35×10^{8} Mc^{-1} s^{-1}	20
6	$NOM + Br_2^{\bullet-} \rightarrow$ products	6.65×10^{5} Mc^{-1} s^{-1}	20
7	$NOM + HOCl \rightarrow$ products	1.50 Mc^{-1} s^{-1}	17
8	$NOM + HOBr \rightarrow$ products	15.00 Mc^{-1} s^{-1}	17
9	$NOM + CO_3^{\bullet-} \rightarrow$ products	3.36×10^{6} Mc^{-1} s^{-1}	17
	**Probe Compounds (6 reactions)**		
1	$NB + HO^{\bullet} \rightarrow$ products	3.90×10^{9} M^{-1} s^{-1}	8
2	$DEET + HO^{\bullet} \rightarrow$ products	6.70×10^{9} M^{-1} s^{-1}	21

3	NPX + $HO^{\bullet}$ → products	8.61×10^{9} M^{-1} s^{-1}	[21]
4	NPX + $Cl^{\bullet}$ → products	2.01×10^{10} M^{-1} s^{-1}	[21]
5	NPX + $Cl_2^{\bullet-}$ → products	6.57×10^{8} M^{-1} s^{-1}	[21]
6	NPX + $CO_3^{\bullet-}$ → products	5.60×10^{7} M^{-1} s^{-1}	[8]

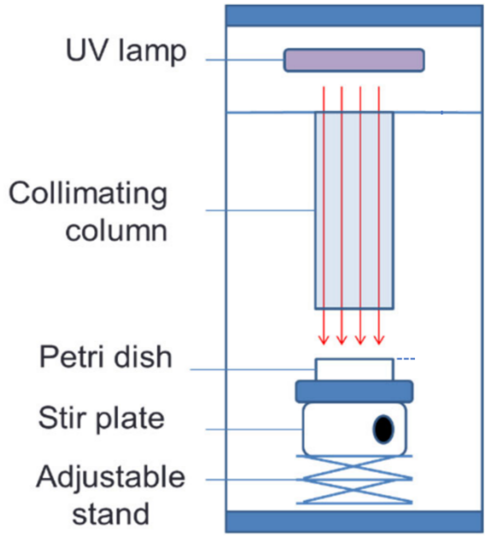

Figure S1. Set-up of the UV reactor in this study.

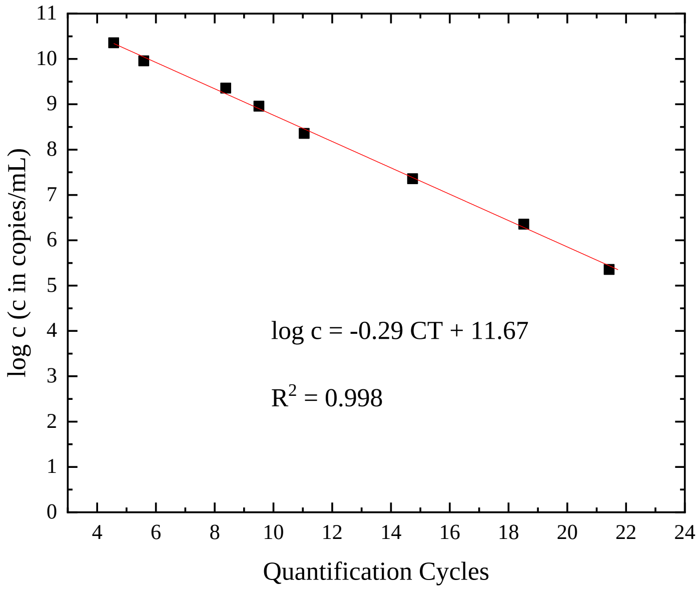

Figure S2. Standard curve for qPCR measurement calibrated from *bla*$_{TEM-1}$ in plasmid pBR322 (Sigma-Aldrich) at concentrations reported by manufacturer.

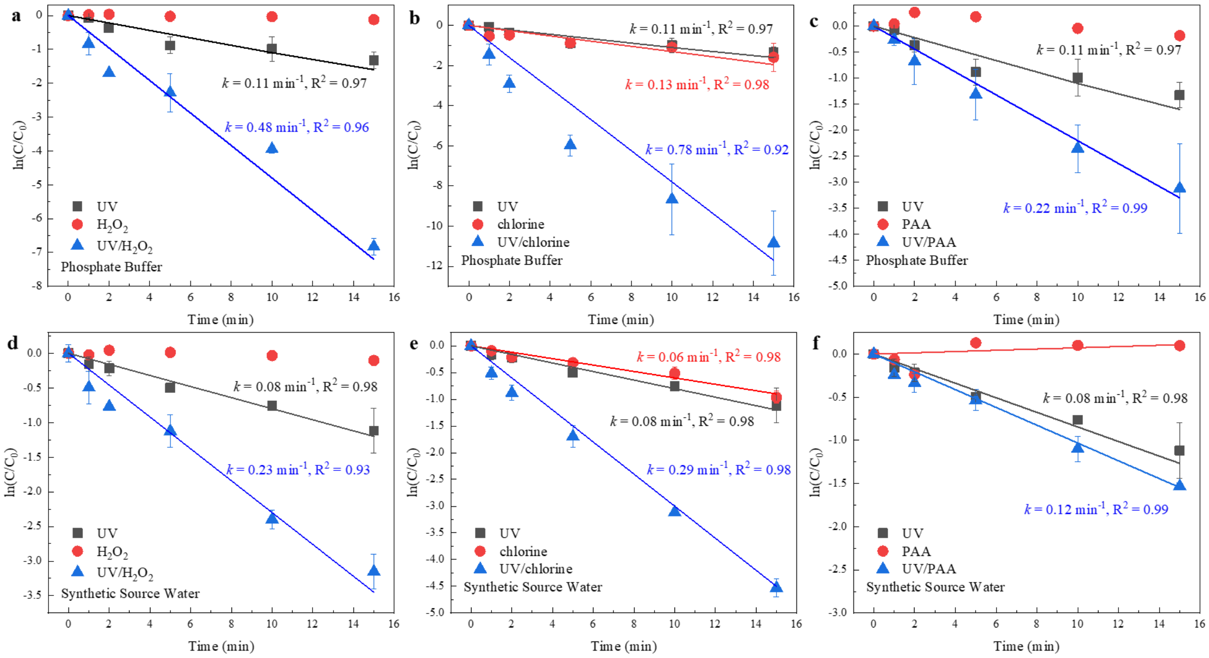

Figure S3. Degradation of eARG by UV/H_2O_2 (a, d), UV/HOCl (b, e), and UV/PAA (c, f) in 10 mM phosphate buffer (upper panels) and synthetic source water matrix (lower panels). Experimental conditions: $[\text{oxidants}]_0 = 50$ µM (50 µM PAA contains 20 µM coexistent H_2O_2), UV fluence rate = 9.5 ± 0.5 Einstein/(L·s) = 0.54 ± 0.03 mW/cm^2, $[\text{eARG}]_0$ = ~1×10^{10} copies/mL, pH = 7.1, temperature = 23 ± 2 °C. Error bars represent standard deviation between duplicate experiments. Solid lines represent linear regression of the results (the data for H_2O_2 and PAA only were not analyzed due to negligible degradation).

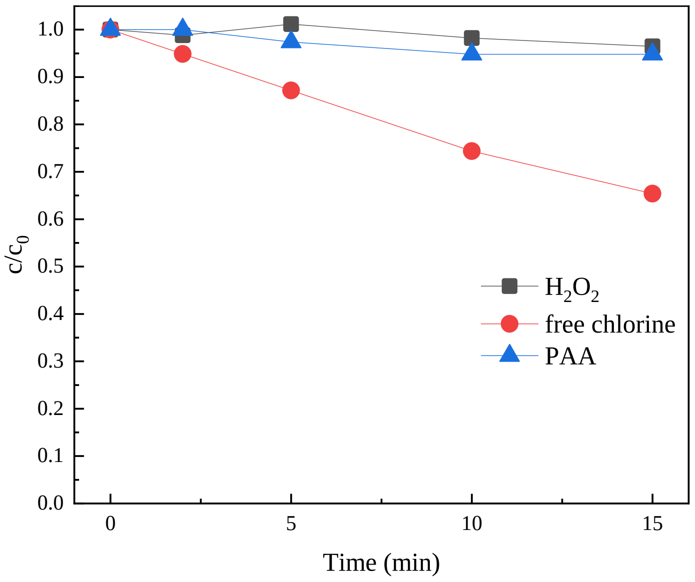

Figure S4. Oxidant loss during iARG degradation experiments. Experimental conditions: $[\text{oxidants}]_0 = 50$ μM (50 μM PAA contains 20 μM coexistent H_2O_2), $[\text{cells}]_0 = {\sim}5\times10^7$ CFU/mL, pH = 7.1, [phosphate buffer] = 10 mM, temperature = 23 ± 2 °C, no UV was applied.

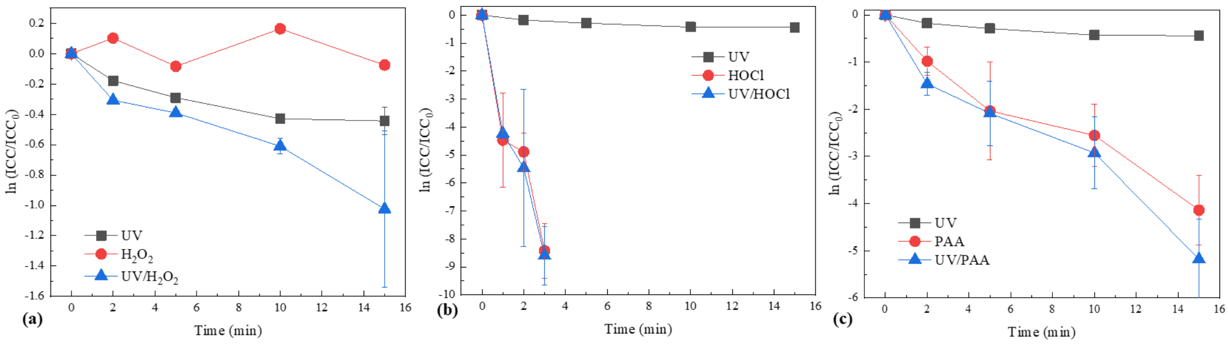

Figure S5. Degradation of intact cell counts (ICCs) by UV/H_2O_2 (a), UV/HOCl (b), and UV/PAA (c) of *E. coli*. Experimental conditions: [oxidants]$_0$ = 50 µM (50 µM PAA contains 20 µM coexistent H_2O_2), [cells]$_0$ = ~5×10^7 CFU/mL, UV fluence rate = 9.5 ± 0.5 Einstein/(L·s) = 0.54 ± 0.03 mW/cm^2, pH = 7.1, [phosphate buffer] = 10 mM, temperature = 23 ± 2 °C. Error bars represent standard deviation between parallel experiments.

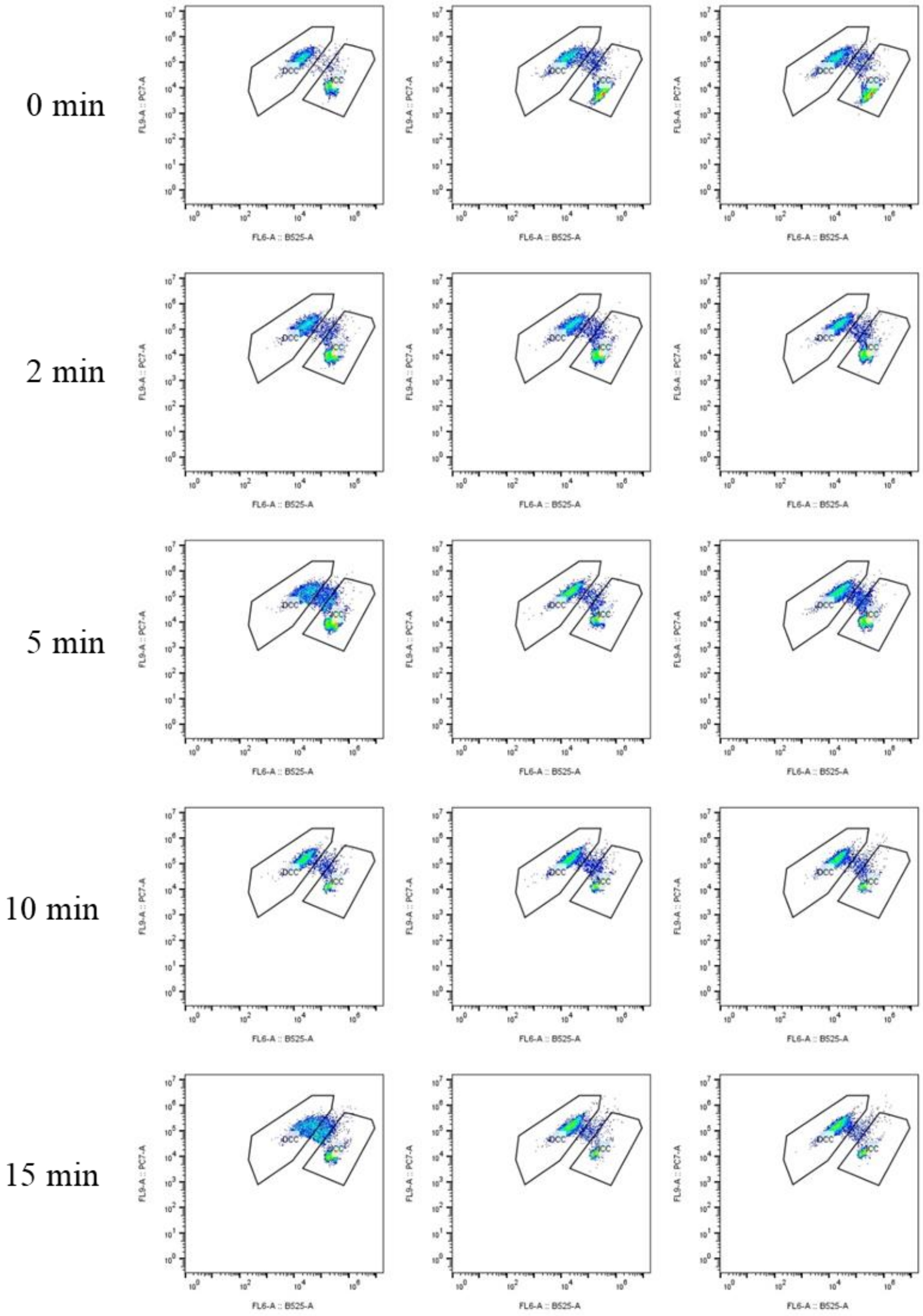

Figure S6. Flow cytometry plots for UV alone treatment of *E. coli* (triplicate results are shown for each time point, conditions see Figure S5).

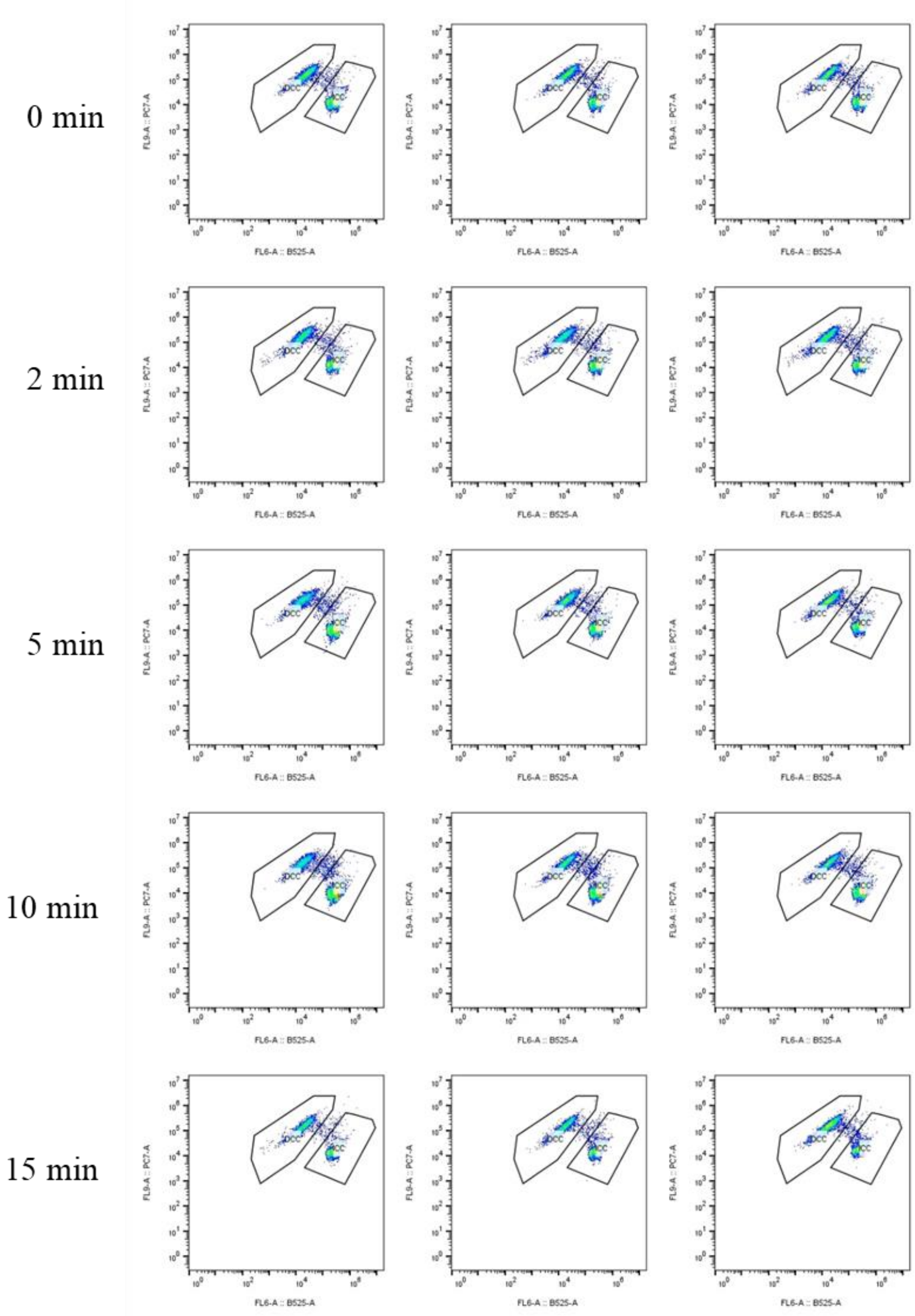

Figure S7. Flow cytometry plots for H_2O_2 alone treatment of *E. coli* (triplicate results are shown for each time point, conditions see Figure S5).

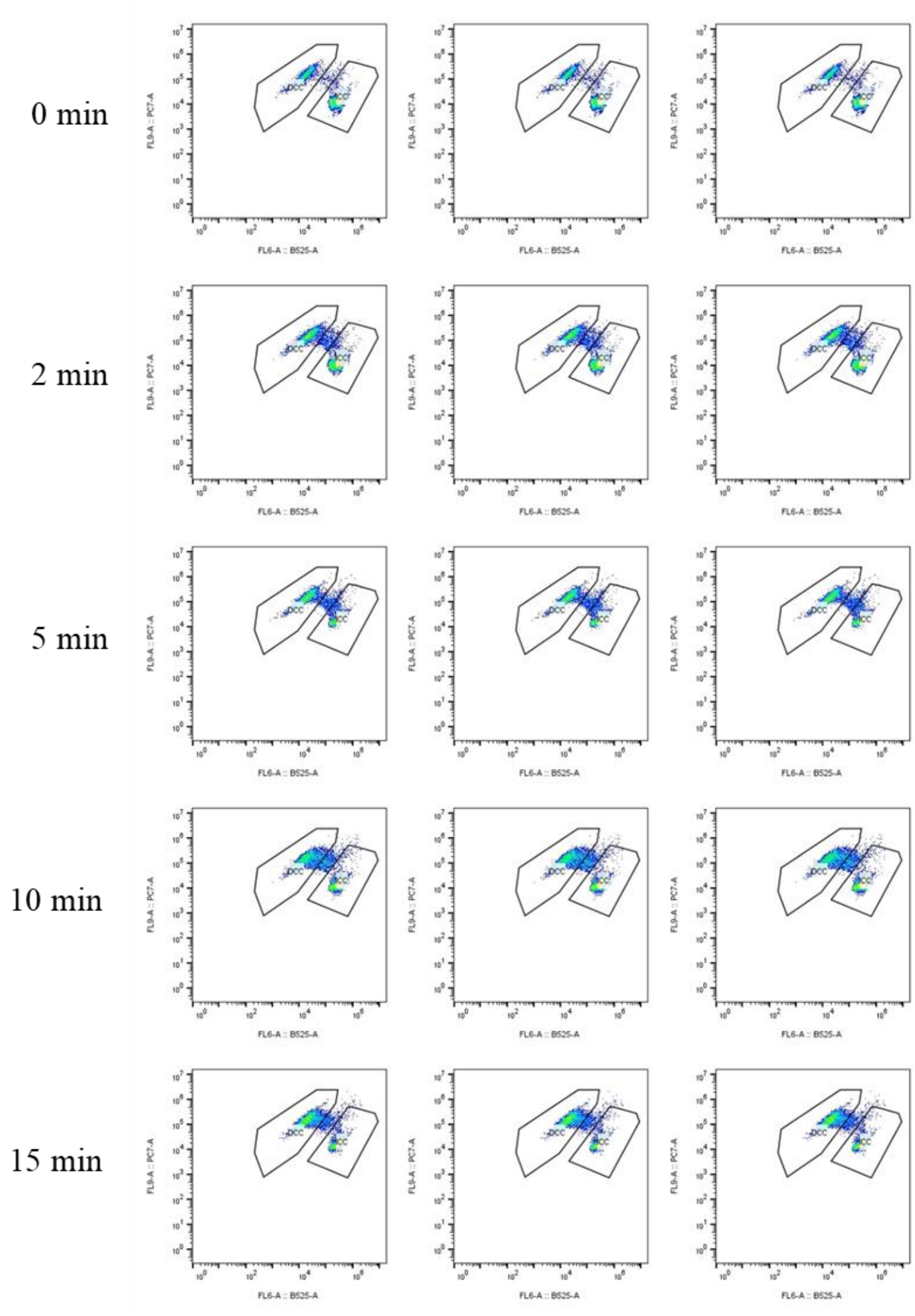

Figure S8. Flow cytometry plots for UV/H_2O_2 treatment of *E. coli* (triplicate results are shown for each time point, conditions see Figure S5).

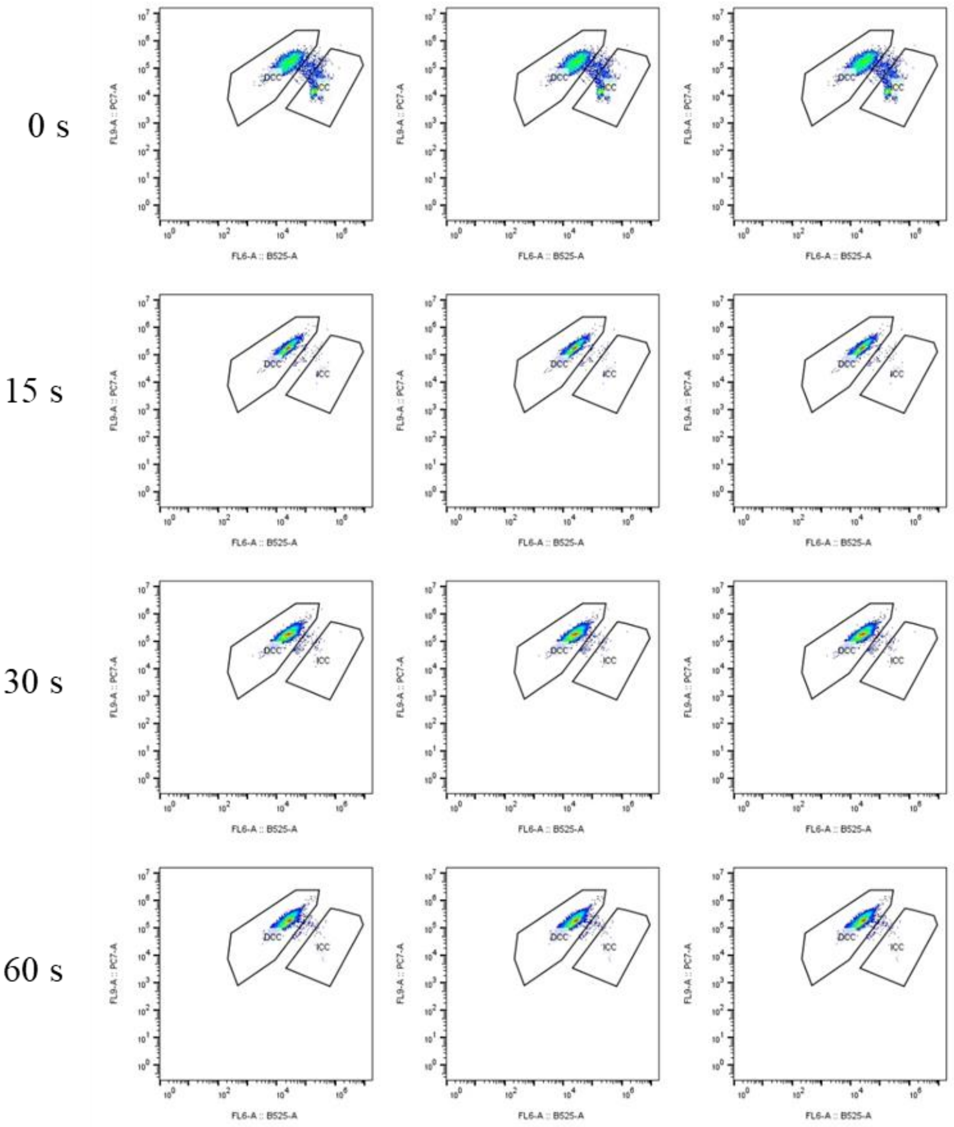

Figure S9. Flow cytometry plots for free chlorine treatment of *E. coli* (triplicate results are shown for each time point, conditions see Figure S5).

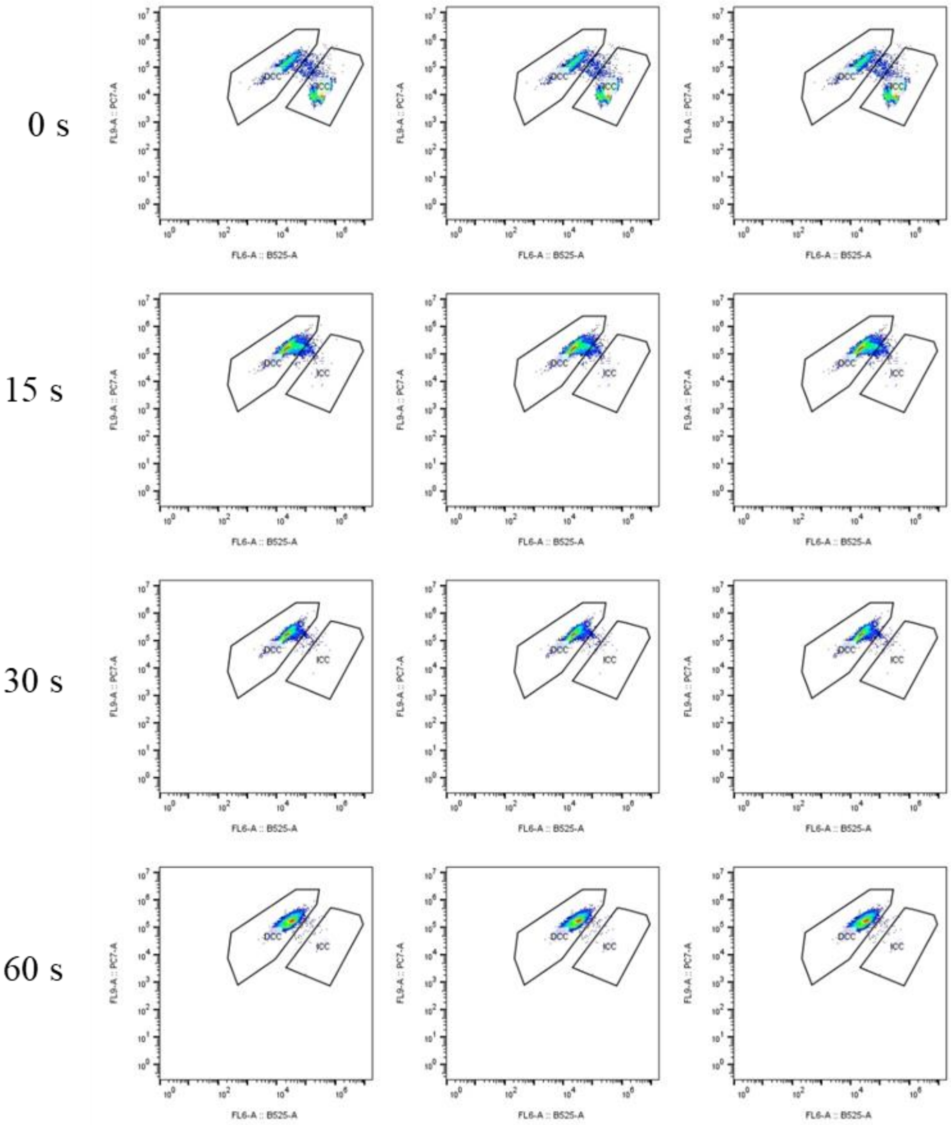

Figure S10. Flow cytometry plots for UV/free chlorine treatment of *E. coli* (triplicate results are shown for each time point, conditions see Figure S5).

0 min

2 min

5 min

10 min

15 min

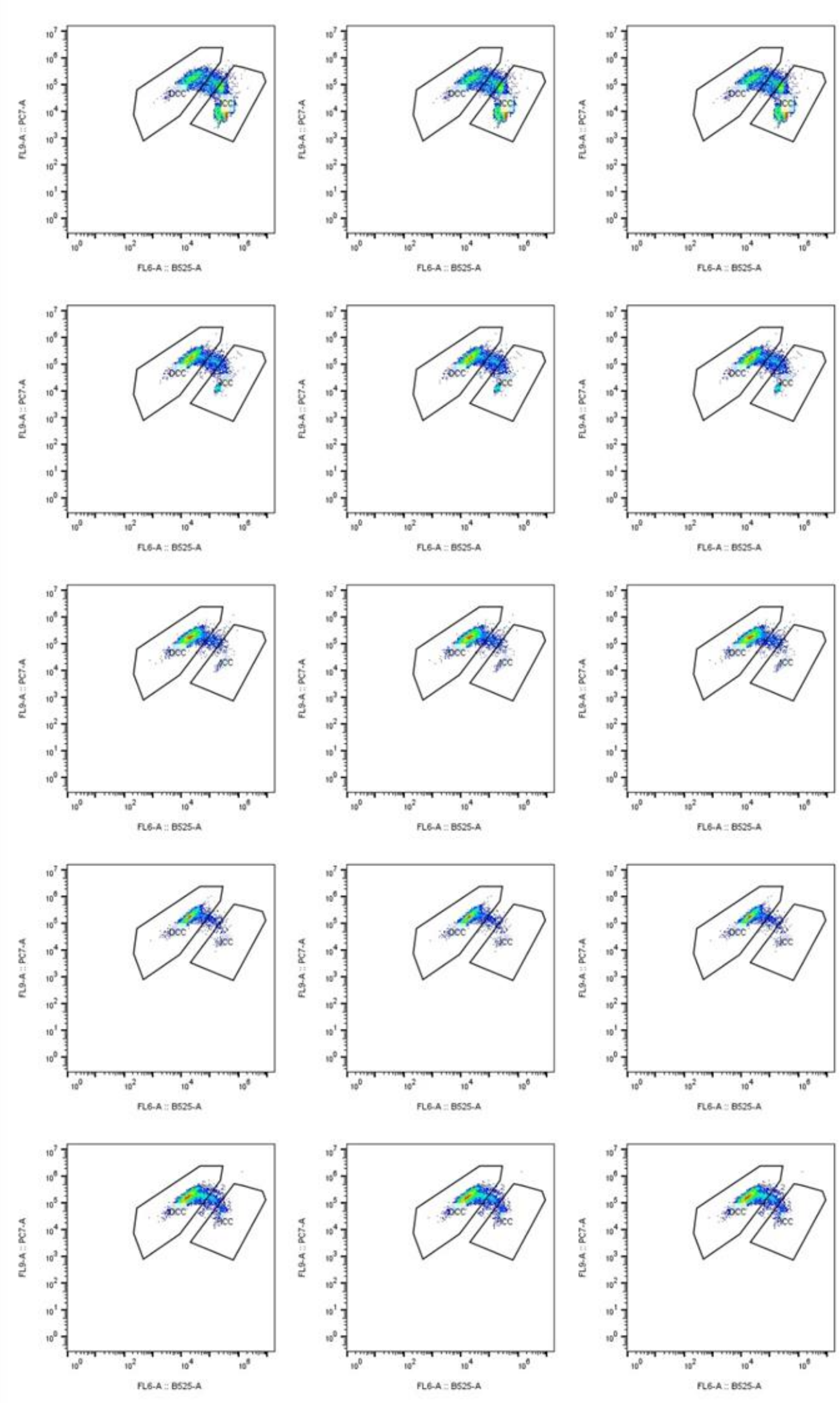

Figure S11. Flow cytometry plots for PAA treatment of *E. coli* (triplicate results are shown for each time point, conditions see Figure S5).

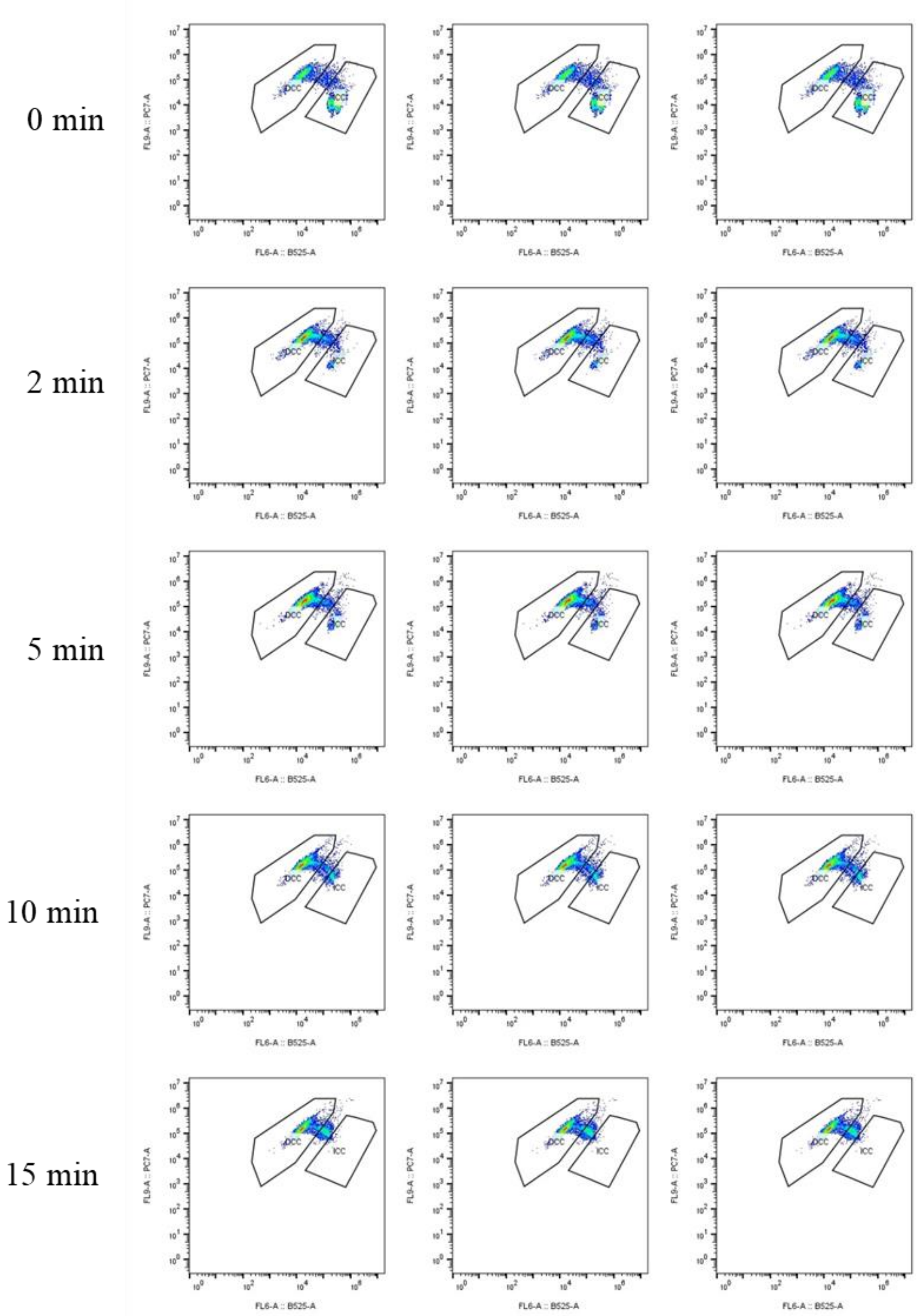

Figure S12. Flow cytometry plots for UV/PAA treatment of *E. coli* (triplicate results are shown for each time point, conditions see Figure S5).

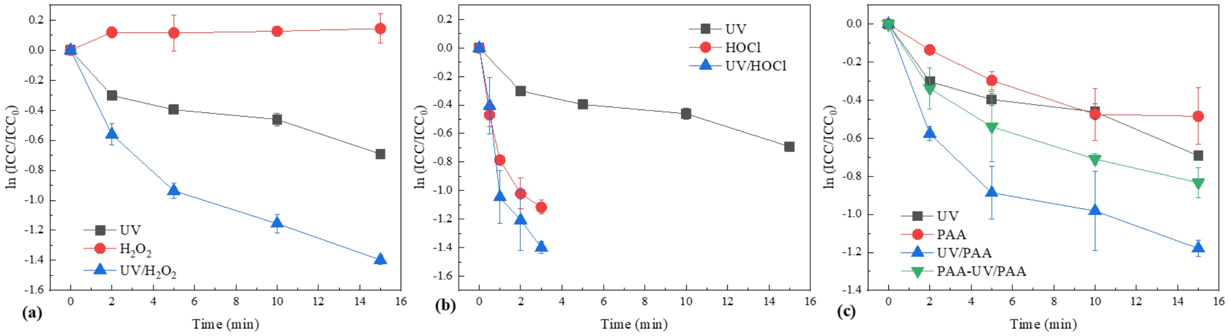

Figure S13. Degradation of intact cell counts (ICCs) by UV/H_2O_2 (a), UV/HOCl (b), and UV/PAA (c) of the bacteria community recovered from bench-scale BAC effluents. Experimental conditions: [oxidants]$_0$ = 50 µM (50 µM PAA contains 20 µM coexistent H_2O_2), UV fluence rate = 9.5 ± 0.5 Einstein/(L·s) = 0.54 ± 0.03 mW/cm^2, pH = 7.1, [phosphate buffer] = 10 mM, temperature = 23 ± 2 °C. Pre-exposure time (to PAA) for PAA-UV/PAA = 2 min. Error bars represent standard deviation between parallel experiments.

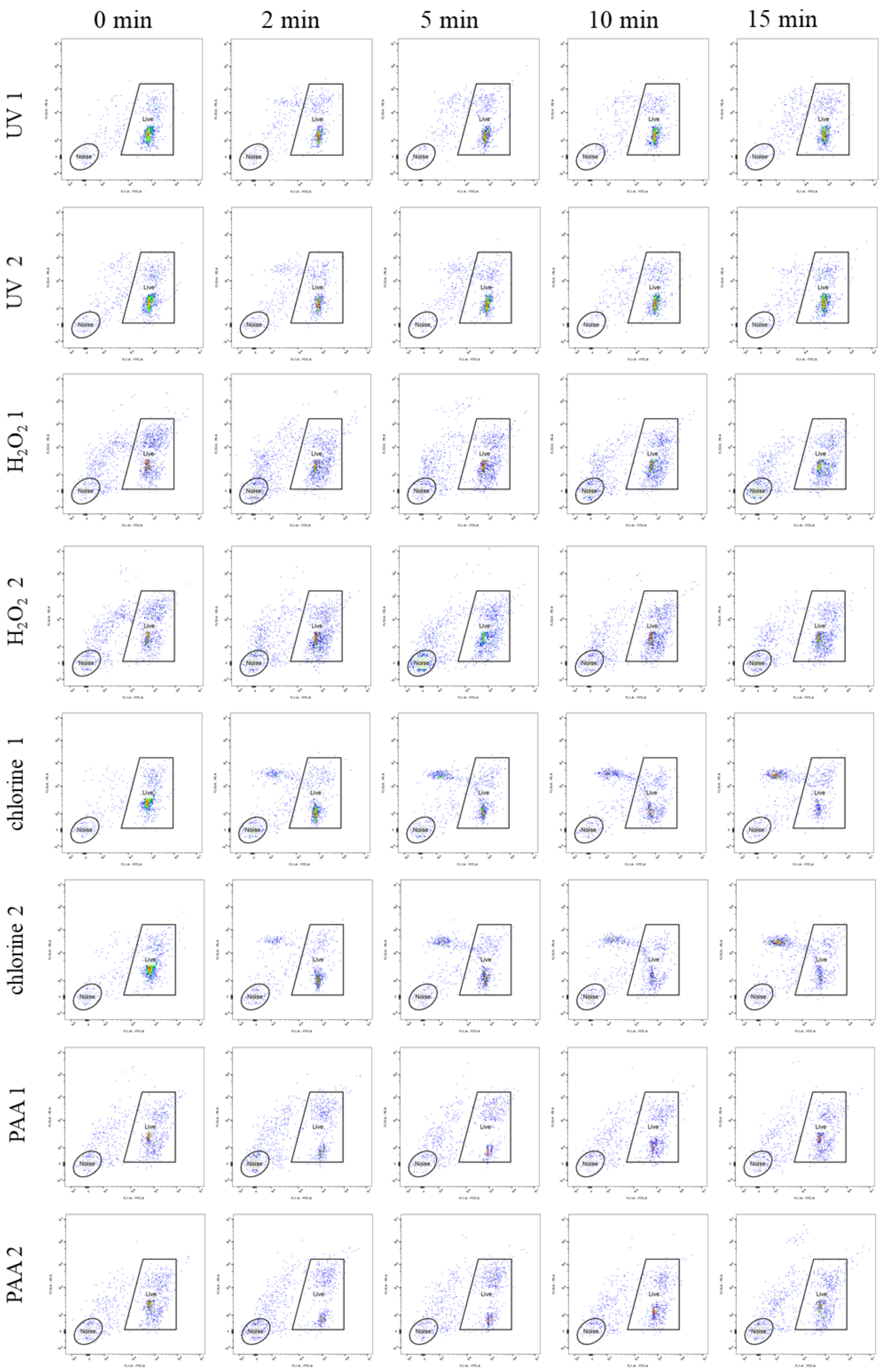

Figure S14. Flow cytometry plots for oxidants or UV alone treatments of BAC bacteria community (duplicate results are shown, conditions see Figure S13).

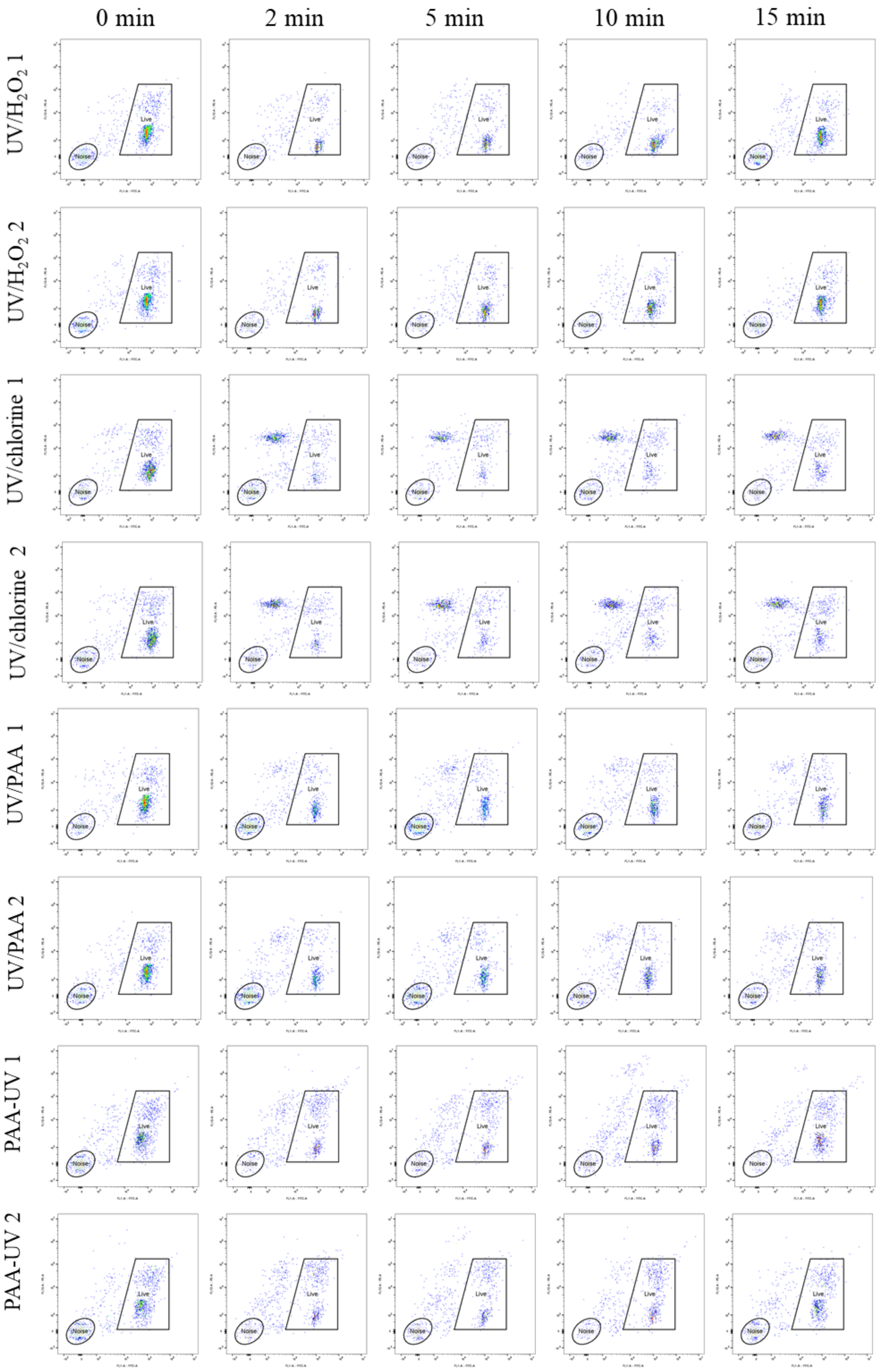

Figure S15. Flow cytometry plots for UV-AOP treatments of BAC bacteria community (duplicate results are shown, conditions see Figure S13).

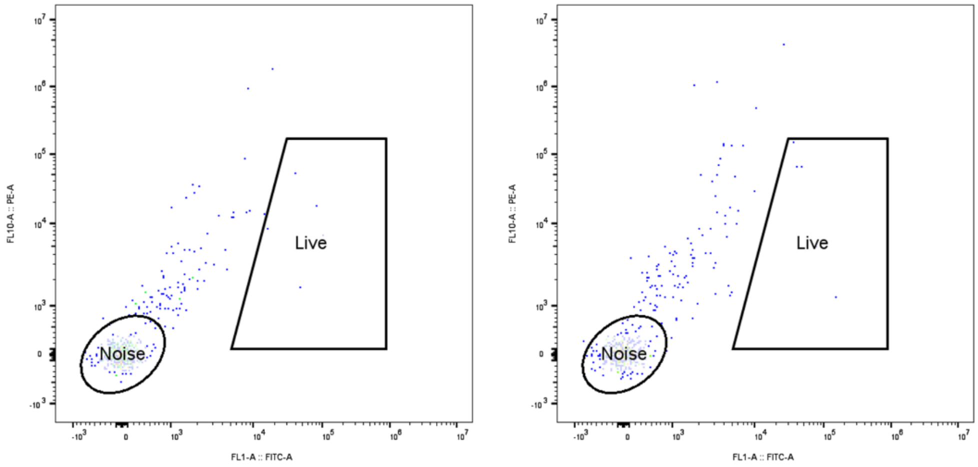

Figure S16. Flow cytometry plots of blank samples (DI water) for determination of the noise gating in BAC community samples.

References

1. Maraccini, P. A.; Mattioli, M. C.; Sassoubre, L. M.; Cao, Y.; Griffith, J. F.; Ervin, J. S.; Van De Werfhorst, L. C.; Boehm, A. B., Solar Inactivation of Enterococci and Escherichia coli in Natural Waters: Effects of Water Absorbance and Depth. *Environ. Sci. Technol.* **2016,** *50*, (10), 5068-76.
2. Mwatondo, M. H.; Silverman, A. I., Escherichia coli and Enterococcus spp. Indigenous to Wastewater Have Slower Free Chlorine Disinfection Rates than Their Laboratory-Cultured Counterparts. *Environ. Sci. Technol. Lett.* **2021,** *8*, (12), 1091-1097.
3. Wang, J.; Chen, W.; Wang, T.; Reid, E.; Krall, C.; Kim, J.; Zhang, T.; Xie, X.; Huang, C.-H., Bacteria and Virus Inactivation: Relative Efficacy and Mechanisms of Peroxyacids and Chlor(am)ine. *Environ. Sci. Technol.* **2023**.
4. Bairoliya, S.; Koh Zhi Xiang, J.; Cao, B., Extracellular DNA in Environmental Samples: Occurrence, Extraction, Quantification, and Impact on Microbial Biodiversity Assessment. *Appl. Environ. Microbio.* **2022,** *88*, (3), e01845-21.
5. Choi, Y.; He, H.; Dodd, M. C.; Lee, Y., Degradation Kinetics of Antibiotic Resistance Gene mecA of Methicillin-Resistant Staphylococcus aureus (MRSA) during Water Disinfection with Chlorine, Ozone, and Ultraviolet Light. *Environ. Sci. Technol.* **2021,** *55*, (4), 2541-2552.
6. Johnson, G.; Nour, A. A.; Nolan, T.; Huggett, J.; Bustin, S., Minimum Information Necessary for Quantitative Real-Time PCR Experiments. In *Quantitative Real-Time PCR: Methods and Protocols*, Biassoni, R.; Raso, A., Eds. Springer New York: New York, NY, 2014; pp 5-17.
7. He, H.; Choi, Y.; Wu, S. J.; Fang, X.; Anderson, A. K.; Liou, S. Y.; Roberts, M. C.; Lee, Y.; Dodd, M. C., Application of Nucleotide-Based Kinetic Modeling Approaches to Predict Antibiotic Resistance Gene Degradation during UV- and Chlorine-Based Wastewater Disinfection Processes: From Bench- to Full-Scale. *Environ. Sci. Technol.* **2022,** *56*, (21), 15141-15155.
8. Guo, K.; Wu, Z.; Yan, S.; Yao, B.; Song, W.; Hua, Z.; Zhang, X.; Kong, X.; Li, X.; Fang, J., Comparison of the UV/chlorine and UV/H_2O_2 processes in the degradation of PPCPs in simulated drinking water and wastewater: Kinetics, radical mechanism and energy requirements. *Water Res.* **2018,** *147*, 184-194.
9. Bulman, D. M.; Mezyk, S. P.; Remucal, C. K., The Impact of pH and Irradiation Wavelength on the Production of Reactive Oxidants during Chlorine Photolysis. *Environ. Sci. Technol.* **2019,** *53*, (8), 4450-4459.
10. Zhang, Z.; Chuang, Y. H.; Szczuka, A.; Ishida, K. P.; Roback, S.; Plumlee, M. H.; Mitch, W. A., Pilot-scale evaluation of oxidant speciation, 1,4-dioxane degradation and disinfection byproduct formation during UV/hydrogen peroxide, UV/free chlorine and UV/chloramines advanced oxidation process treatment for potable reuse. *Water Res.* **2019,** *164*, 114939.
11. Chen, C.; Wu, Z.; Zheng, S.; Wang, L.; Niu, X.; Fang, J., Comparative Study for Interactions of Sulfate Radical and Hydroxyl Radical with Phenol in the Presence of Nitrite. *Environ. Sci. Technol.* **2020,** *54*, (13), 8455-8463.
12. Li, W.; Patton, S.; Gleason, J. M.; Mezyk, S. P.; Ishida, K. P.; Liu, H., UV Photolysis of Chloramine and Persulfate for 1,4-Dioxane Removal in Reverse-Osmosis Permeate for Potable Water Reuse. *Environ. Sci. Technol.* **2018,** *52*, (11), 6417-6425.
13. Zhang, X.; Ren, P.; Li, W.; Lei, Y.; Yang, X.; Blatchley, E. R., 3rd, Synergistic removal of ammonium by monochloramine photolysis. *Water Res.* **2019,** *152*, 226-233.
14. Lian, L.; Yao, B.; Hou, S.; Fang, J.; Yan, S.; Song, W., Kinetic Study of Hydroxyl and Sulfate Radical-Mediated Oxidation of Pharmaceuticals in Wastewater Effluents. *Environ. Sci. Technol.* **2017,** *51*, (5), 2954-2962.

15. Zhang, R.; Yang, Y.; Huang, C.-H.; Li, N.; Liu, H.; Zhao, L.; Sun, P., UV/H_2O_2 and UV/PDS Treatment of Trimethoprim and Sulfamethoxazole in Synthetic Human Urine: Transformation Products and Toxicity. *Environ. Sci. Technol.* **2016,** *50*, (5), 2573-2583.
16. Zhang, T.; Huang, C.-H., Modeling the Kinetics of UV/Peracetic Acid Advanced Oxidation Process. *Environ. Sci. Technol.* **2020,** *54*, (12), 7579-7590.
17. Guo, K.; Zheng, S.; Zhang, X.; Zhao, L.; Ji, S.; Chen, C.; Wu, Z.; Wang, D.; Fang, J., Roles of Bromine Radicals and Hydroxyl Radicals in the Degradation of Micropollutants by the UV/Bromine Process. *Environ. Sci. Technol.* **2020,** *54*, (10), 6415-6426.
18. Zhai, H.; Zhang, X.; Zhu, X.; Liu, J.; Ji, M., Formation of brominated disinfection byproducts during Chloramination of drinking water: new polar species and overall kinetics. *Environ. Sci. Technol.* **2014,** *48*, (5), 2579-88.
19. Lei, Y.; Lei, X.; Westerhoff, P.; Zhang, X.; Yang, X., Reactivity of Chlorine Radicals (Cl(*) and Cl_2(*-)) with Dissolved Organic Matter and the Formation of Chlorinated Byproducts. *Environ. Sci. Technol.* **2021,** *55*, (1), 689-699.
20. Lei, Y.; Lei, X.; Westerhoff, P.; Tong, X.; Ren, J.; Zhou, Y.; Cheng, S.; Ouyang, G.; Yang, X., Bromine Radical (Br(*) and Br_2(*-)) Reactivity with Dissolved Organic Matter and Brominated Organic Byproduct Formation. *Environ. Sci. Technol.* **2022,** *56*, (8), 5189-5199.
21. Lei, Y.; Cheng, S.; Luo, N.; Yang, X.; An, T., Rate Constants and Mechanisms of the Reactions of Cl(*) and Cl_2(*-) with Trace Organic Contaminants. *Environ. Sci. Technol.* **2019,** *53*, (19), 11170-11182.
